# Microbial Contamination, Hygienic Practices, and Antimicrobial Resistance Patterns of Food Milling Machines in Somanya, Ghana

**DOI:** 10.1155/ijfo/2474171

**Published:** 2026-06-17

**Authors:** Benjamin Atta Frimpong, Aboagye Kwarteng Dofuor, Seyram Kofi Loh

**Affiliations:** ^1^ Department of Biological Sciences, School of Natural and Environmental Sciences, University of Environment and Sustainable Development, Somanya, Ghana, uesd.edu.gh; ^2^ Department of Built Environment, School of Sustainable Development, University of Environment and Sustainable Development, Somanya, Ghana, uesd.edu.gh

**Keywords:** antimicrobial susceptibility test, bacterial load, contamination, food milling machines, hygiene

## Abstract

The introduction of food milling machines has accelerated food processing, improved time efficiency, and reduced processing costs. Although food safety remains a crucial public health issue, concerns have been raised about potential microbial contamination of these machines due to unhygienic practices in the working environment and on the equipment, which can ultimately contaminate food products. This research is aimed at assessing the microbial contamination, hygienic practices, and antimicrobial resistance patterns associated with food milling machines in Somanya, Yilo Krobo Municipality, in the Eastern Region of Ghana. We employed a descriptive cross‐sectional design, with sampling conducted from food milling machines in selected areas of Somanya. A total of 129 bacterial species, classified into six (6) groups, were identified using MALDI‐TOF. The most prevalent species identified included *Klebsiella* spp. (41.86%), *Enterobacter* spp. (23.25%), *Acinetobacter* spp. (10.08%), *Escherichia coli* (8.53%), *Salmonella* spp. (1.55%), and other species (14.73%). The average bacterial load and coliform count in the swab suspensions were 2.7938 × 10^6^ and 2.3709 × 10^6^ CFU/mL, respectively. Antimicrobial susceptibility testing showed high resistance to ampicillin among *Enterobacter* spp., *Klebsiella* spp., *E. coli*, and *Acinetobacter* spp., while no isolate was resistant to amikacin. The study identified several bacterial species whose presence around these machines raises concerns about hygiene and sanitary practices, calling for stringent hygiene and sanitation regulations among operators and across machines in Somanya, Ghana.

## 1. Introduction

The prevalence of harmful microbes such as bacteria, viruses, and fungi in food products compromises food safety, leading to over 200 diseases from diarrhea to cancer [1]. Over 420,000 foodborne infection–related deaths have been reported annually in underdeveloped and developing countries [2, 3]. A report in 2019 from the World Health Organization (WHO) also revealed that 91 million minor diseases and 137,000 deaths occur in Africa, where children under five are the most vulnerable as a result of food‐related risks.

Food processing chains are contributors to food‐related diseases and deaths [3]. An important step in the food processing chain is milling, the process of pulverizing and concentrating fresh food ingredients into various products, including rice, flour, and other milled grains [4]. Traditional food‐processing tools such as stones, pestles, and mortars are effective but slow and labor‐intensive. This inefficiency led to the development of milling machines, which use rotating grinding discs to rub and pulverize food much faster and have since become the dominant method [2]. During operation, the discs rotate and knead against each other, grinding the food into a powder or paste. Friction from the disc′s movement causes wear and introduces contaminants, such as microbes, into the milled food [5, 6].

Antimicrobial resistance (AMR) has been ranked among the Top 10 worldwide public health dangers and is also a threat to food security [7, 8]. AMR is the ability of microbes, such as viruses, bacteria, fungi, and parasites, to survive and persist in the presence of drugs, including antibiotics, antifungals, and other pharmaceuticals designed to eliminate them [8]. AMR affects countries across all income levels, including high‐income, middle‐income, and low‐income countries. In Ghana, AMR has been estimated to account for 5900 attributable deaths and 25,300 associated deaths [7]. Bacterial resistance to antibiotics is present in humans, animals, water, and the environment and can, therefore, be transferred across these media, as well as through organic manures, including pig and cow manure [8, 9]. These organic manures are used on food crops such as grains, vegetables, legumes, and fruits, and they are irrigated with water that may carry resistant microbes. Resistant microbes attach to food crops and pass through food distribution systems and the food processing chain, such as milling, where they are further spread to consumers.

Somanya is a major commercial town in the Yilo Krobo Municipality, where food milling is commonly used by households and food vendors. With food milling becoming a crucial part of food processing in recent times, food milling operators need to operate under optimal conditions to ensure food safety and be effectively monitored by health and food safety units in municipalities. However, limited data exist on the hygienic status, microbial contamination, and AMR profiles of food milling machines in the area. Thus, there is a knowledge gap on the hygienic status of food milling machines in Somanya. This study is aimed at assessing microbial contamination, hygiene practices, and AMR patterns associated with food milling machines in Somanya, Ghana, in order to generate evidence for improved food safety monitoring and sanitation practices.

## 2. Materials and Methods

### 2.1. Study Area and Site

This research was a descriptive cross‐sectional study in which food milling machines in Somanya were sampled. The study focused on food milling machines and their operators. The Yilo Krobo Municipality, with its administrative capital in Somanya, is a vibrant area in Ghana′s Eastern Region. Spanning 514.7 km^2^, the municipality was home to 122,705 people in 2021 [10]. The municipality is about 80% mountainous, with the Akwapim Range extending from the southwest to northwest [10]. The primary activity in the Yilo Krobo Municipality is agriculture. Somanya is located at 6°06 ^′^14 ^″^ N, 0°00 ^′^54 ^″^ W and serves as a hub of activity.

### 2.2. Point Distribution Map of the Study Area

**Figure 1:**
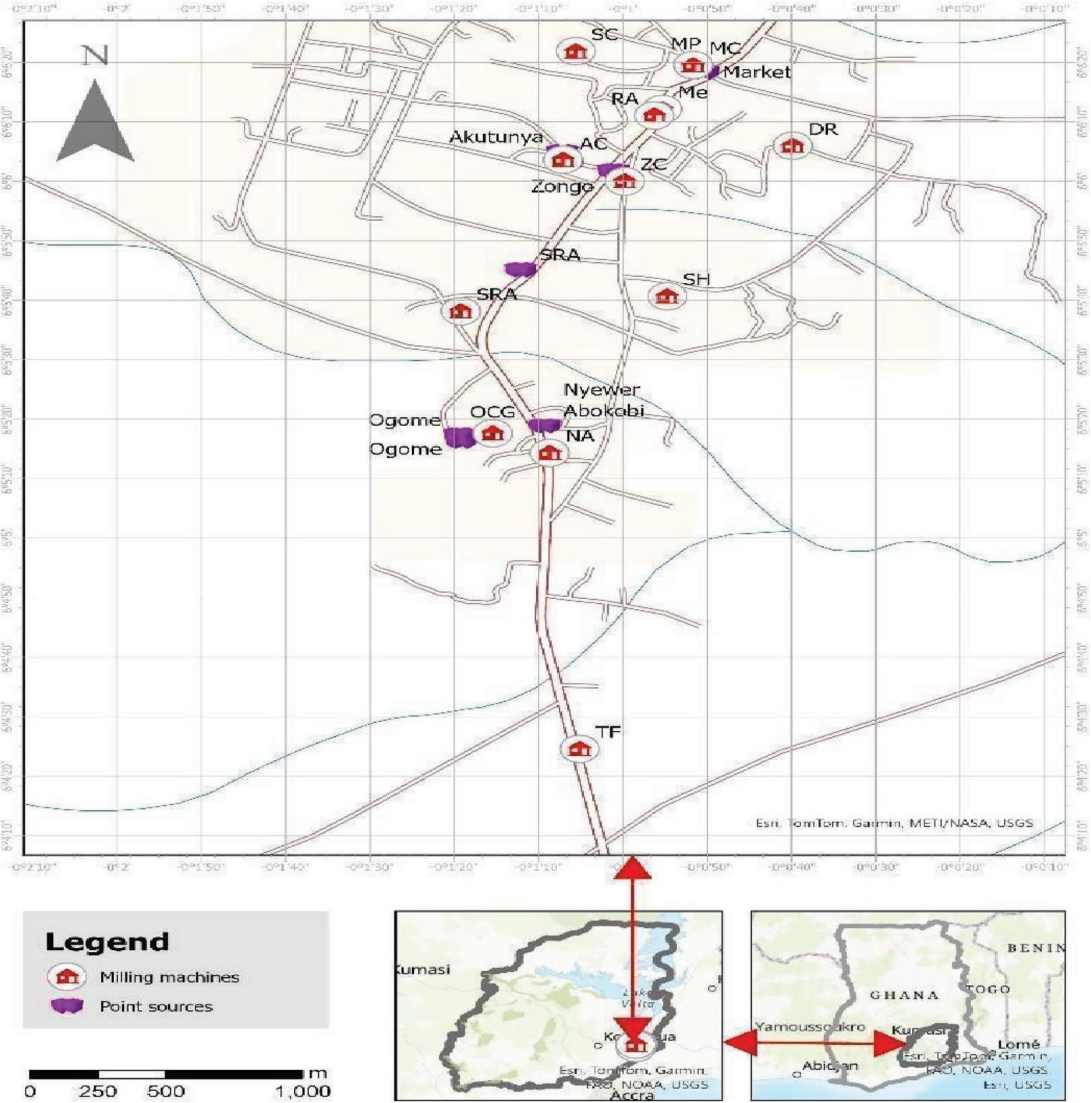
Plot sample locations showing the sampling network and coverage. Each red dot signifies a designated sample site from which bacterial and coliform data were obtained.

The map in Figure 1 depicts the spatial arrangement of sampling locations in the Yilo Krobo Municipality, Ghana. The primary panel displays specific site locations throughout prominent communities, including Somanya town center, Trom, Akorley, and Dzankukaye, with concentrated clusters evident in the middle and northern regions of the entire Somanya township. The map also includes inset panels that provide context for the study region at larger scales, indicating its position within Ghana (top right), the Eastern Region (middle right), and the Yilo Krobo administrative boundary (bottom right).

### 2.3. Microbiological Sampling

Out of 47 milling machines identified in the town, only 34 were functional and operational at the time of the study. By permission of the operators, these 34 functional milling machines were sampled. The nonfunctional machines were excluded from the study. Sampled machines included fufu (16), corn (14), and tomato/pepper milling machines (4) (Table 1). At the sampling site, sterile swab sticks were used to swab the inlet, grinding compartment, and the outlet of the milling machines, making a total of 102 (i.e., 34 × 3) swab samples. Each swab was dipped into peptone water contained in centrifuge tubes and labeled according to the kind of milling machine sampled and the location. Each milling machine was sampled once in the month of June 2025, whereby three swabs were collected from each machine. For instance, if the inlet, outlet, and grinding compartment of a pepper/tomato milling machine were sampled in Ogome, they were labeled OPTI, OPTO, and OPTG, respectively, meaning the Ogome pepper/tomato inlet (I), outlet (O), and grinding compartment (G). Similarly, if the inlet, outlet, and grinding compartment of a fufu milling machine were sampled in Nyewer‐Abokobi, they were labeled NAFI, NAFO, and NAFG, respectively, meaning the Nyewer‐Abokobi fufu inlet (I), outlet (O), and grinding compartment (G). This process was repeated for each sampling site. The bacterial load was expressed as colony‐forming units per milliliter (CFU/mL), representing the concentration of bacteria in the swab suspension. Each swab was suspended in 5 mL of sterile peptone water, vortexed to dislodge surface bacteria, and serially diluted to a factor of 10^−4^ before plating. The bacterial count was calculated using the formula: CFU/mL = (number of colonies counted × dilution factor)/volume plated (mL). As the swabbed surface areas across machine compartments (inlet, grinding compartment, and outlet) were not uniform in geometry and were not measured, results are reported as CFU/mL of suspension rather than CFU/cm^2^, which would require a defined and standardized swab area. This approach is consistent with comparable surface swab studies in food processing environments where suspension‐based counts were used [3, 11].

**Table 1:** Distribution of sampling among the three types of food milling machines.

Sampling site	Tomato/pepper mill	Corn mill	Fufu mill	Total
Trom	—	—	1	1
Ogome	—	2	2	4
Nyewer‐Abokobi	—	1	1	2
Zongo	—	1	—	1
Djaba Road	—	—	1	1
Slaughter house	—	—	1	1
Market	1	1	—	2
Salesu	—	1	1	2
Mount Mary	1	1	1	3
Sawer	2	3	4	9
Sawer‐Quaters	—	1	—	1
Roundabout	—	—	1	1
Methodist school	—	—	2	2
SRA	—	1	1	2
Akutunya Market	—	2	—	2
Total	4	14	16	34

### 2.4. Questionnaires and Interviews

A structured questionnaire containing both closed‐ and open‐ended items was administered to machine operators to gather information on personal hygiene, food safety practices, maintenance of milling machines, and knowledge of microbial contamination (Table S1A,B). Direct observation of the operational practices and environmental conditions around the milling machines was also done.

### 2.5. Microbial Analysis and Identification

Swabs obtained from the various sampling sites were cut into Falcon tubes containing 2 mL of peptone water, vortexed, and incubated for 12–18 h. Swab samples were vortexed again and diluted up to 10^−4^. To determine bacterial load and coliform counts, the various dilutions were cultured on Thermo Scientific Oxoid Nutrient agar (for bacterial load) and MacConkey agar (for coliform count) and incubated for 12–18 h to obtain countable microbial colonies [11]. MacConkey agar was used to support the isolation of Gram‐negative coliform bacteria, which are widely used as indicators of sanitary quality in food processing environments. Their presence suggests possible fecal contamination and inadequate hygiene, which informed the choice of culture media. The colony count for each dilution factor was subsequently tallied, and the average was determined. Cultures on Thermo Scientific Oxoid MacConkey agar were also subcultured onto different MacConkey agars and incubated for 12–18 h. The cultures were purified, and microbial identification was done with the matrix‐assisted laser desorption/ionization time‐of‐flight (MALDI‐TOF) using Bruker MALDI Biotyper 4.1.100 (PYTH) 188 2020‐04‐112_10‐35‐53.

### 2.6. Antimicrobial Susceptibility Test

Using the Clinical and Laboratory Standards Institute [12] guidelines, the susceptibility of pathogenic isolates to standard antimicrobials was tested by the Kirby–Bauer method [13]. Tetracycline (30 *μ*g), cotrimoxazole (1.25 + 23.75 * μ*g), ampicillin (10 *μ*g), cefotaxime (30 *μ*g), amikacin (30 *μ*g), and ciprofloxacin (5 *μ*g), all from Liofilchem, were used in the test. Antibiotic sensitivity was tested using *Escherichia coli* (*E. coli*) ATCC 25922. Each test isolate was emulsified in sterile standard saline solution to create a suspension with turbidity equivalent to a 0.5 McFarland standard using a nephelometer. A sterile cotton swab was dipped into the suspension and pressed against the interior walls of the container to drain excess fluid. It was swabbed evenly across the entire surface of a Thermo Scientific Oxoid Mueller–Hinton agar plate in three dimensions to obtain a semiconfluent growth following incubation. The plates were incubated at 37°C for 18–24 h, after which the zones of inhibition around the antimicrobial discs were measured and interpreted according to the CLSI breakpoints.

### 2.7. Data Analysis

The total number of samples from each sampling site was keyed into Google Sheets, and the results were analyzed using R software Version 4.1.3. The mean bacterial load and mean coliform counts for each milling machine type (i.e., corn mill, tomato/pepper mill, and fufu mill) were calculated. All samples were grouped by machine type, and the mean bacterial load and coliform count were calculated for each group. One‐way ANOVA was performed to compare variation in bacterial load and coliform counts. Chi‐square analysis was also performed to assess the association between hygiene practices and contamination levels.

## 3. Results

### 3.1. Microbial Load and Total Coliform Counts From Food Milling Machines

Table 2 summarizes the mean bacterial load in CFU/mL, with the highest recorded from fufu milling machines, followed by pepper/tomato and corn milling machines, respectively. The bacterial load across the different milling machine types was compared using one‐way ANOVA, which revealed no statistically significant difference (*F* = 0.57, *p* = 0.58).

**Table 2:** Mean bacterial load of tomato/pepper, fufu, and corn machines in Somanya.

Type of milling machine	Sample size	*M* *e* *a* *n* × 10^6^ ± *S* *D*(CFU/mL)	Mean log (CFU/mL)
TPM	4	2.3668 ± 1.972487	6.1087
FMM	16	3.0808 ± 1.696730	6.2947
CMM	14	2.9338 ± 1.705560	6.2743

*Note:* One‐way ANOVA showed no significant difference in mean bacterial load among machine types (*F* = 0.57, *p* = 0.58).

Abbreviations: CFU/mL, colony‐forming unit per milliliter; CMM, corn milling machine; FMM, fufu milling machine; TPM, tomato/pepper machine.

### 3.2. Proportional Symbols by Bacterial Load

**Figure 2:**
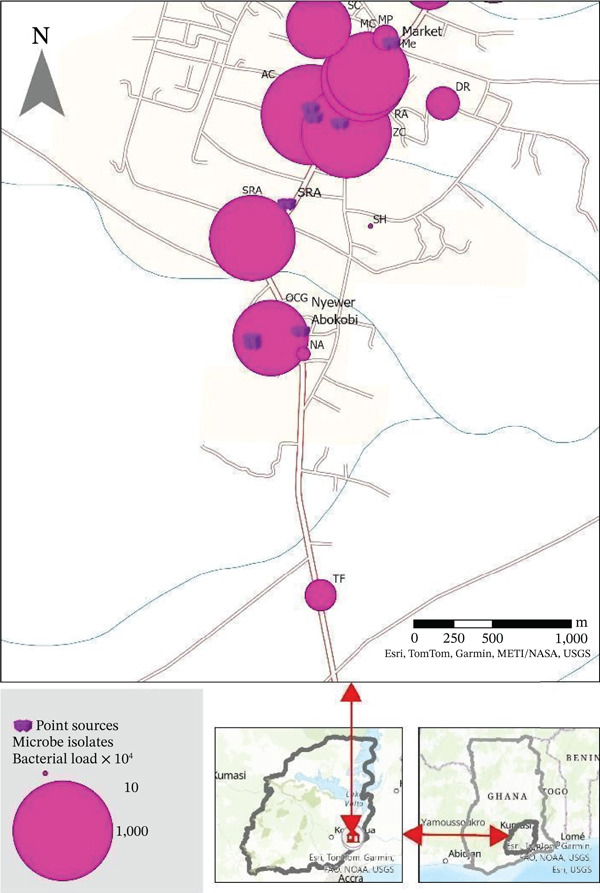
Bacterial load count symbol map. Each red circle denotes a sampling point, with the circle′s size corresponding to the level of bacterial load recorded at that location.

The spatial variation in bacterial contamination levels at sampling sites within the study area was mapped (Figure 2). The map indicates that the highest bacterial concentrations are in the middle and northern regions of the municipality, such as Somanya town center, Sra, and Ogome, areas marked by high levels of human activity and market operations, whereas moderate bacterial loads are present in Trom. The inset maps delineate the study area′s position within the Somanya township and part of the Yilo Krobo Municipality, thereby offering geographic context. The distribution pattern indicates that human activities, such as waste management and proximity to urban areas, may be affecting the changes in bacterial load throughout the municipality.

Table 3 summarizes the average coliform count (CFU/mL) at a dilution factor of 10^4^. The mean average coliform count in corn milling machines was the highest, followed by tomato/pepper and fufu milling machines, respectively. There was no statistically significant difference in the coliform counts across the milling machine types (*F* = 0.71, *p* = 0.494).

**Table 3:** Mean coliform count of tomato/pepper, fufu, and corn machines in Somanya.

Type of milling machine	Sample size	*M* *e* *a* *n* × 10^6^ ± *S* *D*(CFU/mL)	Mean log (CFU/mL)
TPM	4	2.6083 ± 1.8520	6.0778
FMM	16	2.0050 ± 1.8844	5.8982
CMM	14	2.4995 ± 1.8344	6.0703

*Note:* One‐way ANOVA showed no significant difference in mean coliform count among machine types (*F* = 0.71, *p* = 0.494).

Abbreviations: CFU/mL, colony‐forming unit per milliliter; CMM, corn milling machine; FMM, fufu milling machine; TPM, tomato/pepper machine.

### 3.3. Proportional Symbols by Coliform Count

**Figure 3:**
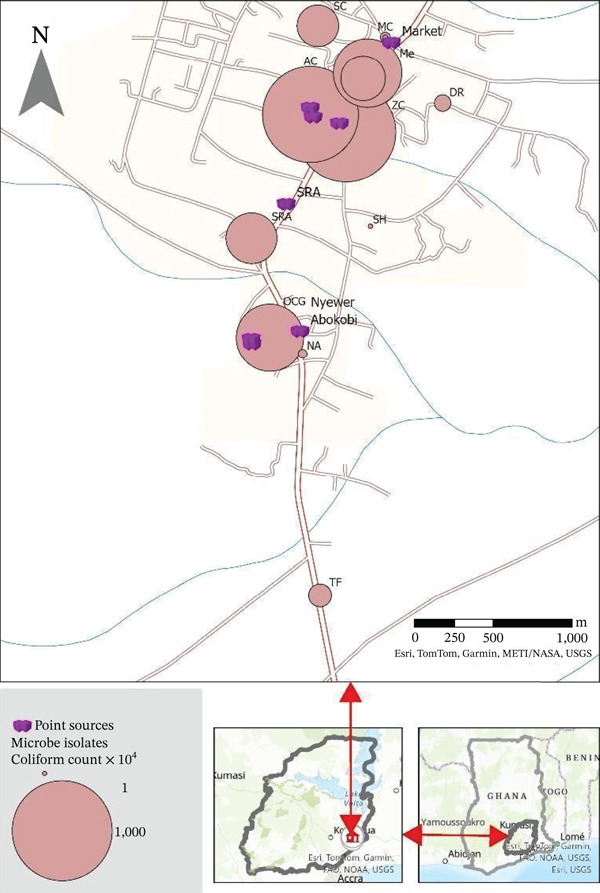
Coliform count symbol map. Each red circle denotes a sampling location, with the size of the symbol corresponding to the quantified coliform concentration.

The spatial variations in coliform contamination counts at sampling sites within Somanya township were mapped (Figure 3). Larger circles, signifying elevated contamination levels, are primarily clustered in Somanya town center, Sra, and Ogome, indicating potential localized sources of pollution in densely populated, market‐driven regions. Smaller circles in the southern settlements, including Trom, exhibit somewhat lower coliform levels. This underscores the necessity for focused management of water and environmental quality in high‐risk areas.

### 3.4. Chi‐Square Analysis of Hygiene Practices and Contamination Levels

As shown in Table 4, washing machine with soap (WMWS) and cleaning frequency were significantly associated with contamination levels (*p* < 0.05), whereas hand hygiene showed no significant association (*p* > 0.05). Since some cells had low expected frequencies, Fisher′s exact test was used to confirm the associations. The distribution of contamination levels across the different hygiene practices is presented in Table 5.

**Table 4:** Association between hygiene practices and contamination level.

Hygiene practice	*χ* ^2^	*d* *f*	Chi‐square *p* value	Fisher *p* value	Interpretation
Washing machine with soap (WMWS)	9.23	2	0.0099	0.0075	Significant
Hand hygiene	1.72	2	0.422	0.4948	Not significant
Cleaning frequency	6.30	2	0.0428	0.0305	Significant

**Table 5:** Distribution of contamination levels by hygiene practice.

Study variable/score	Moderately high	High	Very high
Washing machine with soap			
Yes	3	8	9
No	2	12	0
Hand hygiene			
Yes	2	14	5
No	3	6	4
Cleaning frequency			
Once	2	13	9
Twice	3	7	0

*Note:* Moderately high = less than 1,000,000 (<1 × 10^6^) CFU/mL; High = greater than 1,000,000 and less than 3,000,000 (1 × 10^6^ < *x* < 3 × 10^6^) CFU/mL; Very high = greater than 3,000,000 (>3 × 10^6^) CFU/mL.

### 3.5. Isolated Microbes From Food Milling Machines in a Selected Area in Somanya

**Figure 4:**
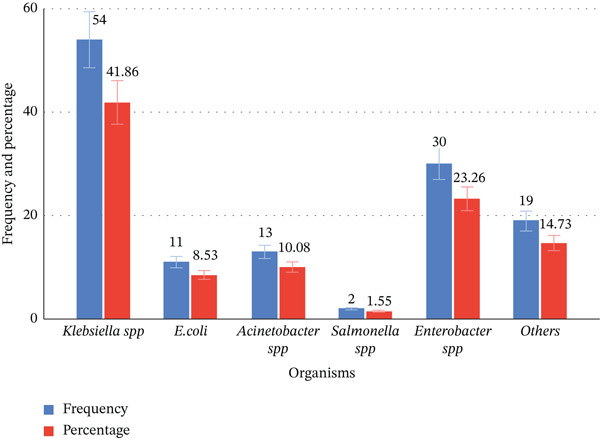
Graphical representation of isolated microbes from food milling machines in selected areas in Somanya. Organisms indicated as “others” included *Pseudomonas* spp., *Citrobacter* spp., Pantoea spp., *Aeromonas* spp., *Cronobacter* spp., and *Mixta calida.*

Figure 4 shows the various bacterial groups isolated from all food milling machines sampled during the study. Out of the 102 swabs sampled from the 34 milling machines, microbial analysis and identification using MALDI‐TOF revealed the presence of 129 bacterial species categorized into six (6) groups of bacteria isolated from the sampled milling stations (Table S2, Figure 4). These isolates included *Klebsiella* spp., *E. coli*, *Acinetobacter* spp., *Salmonella* spp., *Enterobacter* spp., and other microbes (*Citrobacter* spp., *Pantoea* spp., *Pseudomonas* spp., *Aeromonas* spp., *Cronobacter* sp., and *Mixta calida*) (Table S2, Figure 4). *Klebsiella* spp. was the most common bacterial species, followed by *Enterobacter* spp., *Acinetobacter* spp., *E. coli*, and *Salmonella* spp. (Figure 4).

### 3.6. Type of Milling Machine and the Isolated Microbes

Table 6 shows the distribution of bacterial isolates across food milling machines. A total of 129 bacterial isolates were identified from the various food milling machine types, with fufu milling machines having the highest contamination. *Klebsiella* spp. was the most dominant bacterial species isolated, particularly in fufu milling machines, along with *Enterobacter* spp., *Acinetobacter* spp., *E. coli*, and *Salmonella* spp., respectively.

**Table 6:** Frequency of isolated bacteria from the various food milling machine types.

Type of milling machine	*Klebsiella* spp.	*E. coli*	*Acinetobacter* spp.	*Salmonella* spp.	*Enterobacter* spp.	Others	Total
TPM	7 (5.43%)	2 (1.55%)	0 (0%)	0 (0%)	3 (2.33%)	6 (4.65%)	18 (13.95%)
CMM	18 (13.95%)	7 (5.43%)	5 (3.88%)	0 (0%)	13 (10.08%)	9 (6.98%)	52 (40.31%)
FMM	29 (22.48%)	2 (1.55%)	8 (6.20%)	2 (1.55%)	14 (10.85%)	4 (3.10%)	59 (45.74%)
Total	54 (41.86%)	11 (8.53%)	13 (10.08%)	2 (1.55%)	30 (23.26%)	19 (14.73%)	129 (100%)

Abbreviations: CMM, corn milling machine; FMM, fufu milling machine; TPM, tomato/pepper machine.

### 3.7. Isolated Bacteria From the Various Compartments of the Food Milling Machines Sampled During the Study

Table 7 shows the frequency of bacterial isolates recovered from the inlet, outlet, and grinding compartments of the milling machines. It also shows enormous microbial contamination within all compartments of the milling machines.

**Table 7:** Bacterial isolates from the various compartments of food milling machines.

Isolate	Inlet	Outlet	Grinding compartment	Total
*Klebsiella* spp.	21 (16.28%)	15 (11.63%)	18 (13.95%)	54 (41.86%)
*Enterobacter* spp.	5 (3.88%)	14 (10.85%)	11 (8.53%)	30 (23.26%)
*Acinetobacter* spp.	6 (4.65%)	0 (0%)	7 (5.43%)	13 (10.08%)
*E. coli*	2 (1.55%)	4 (3.10%)	5 (3.88%)	11 (8.53%)
*Salmonella* spp.	0 (0%)	1 (0.78%)	1 (0.78%)	2 (1.55%)
Others	7 (5.43%)	6 (4.65%)	6 (4.65%)	19 (14.73%)
Total	41 (31.78%)	40 (31.01%)	48 (37.21%)	129 (100%)

*Note:* Organisms indicated as “others” included *Pseudomonas* spp., *Citrobacter* spp., *Pantoea* spp., *Aeromonas* spp., *Cronobacter* spp., and *Mixta calida.*

### 3.8. Weighted Overlay Analysis (Contamination Risk Mapping)

**Figure 5:**
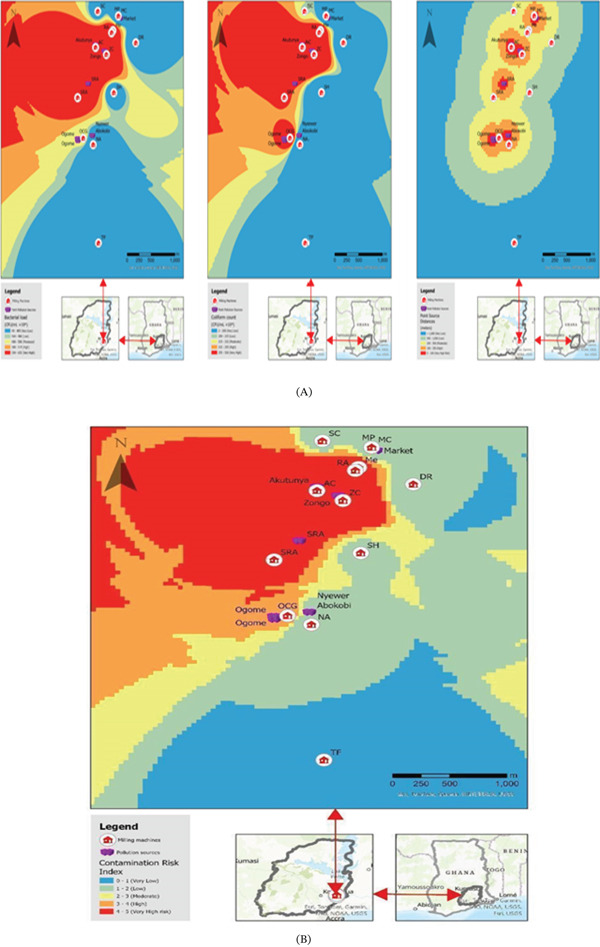
(A) Weighted overlay analysis showing bacterial load, coliform concentration, and proximity to pollution sources. (B) Contamination risk index map for the study area.

A weighted overlay analysis, a technique used for combining several raster layers (grids) where each layer is assigned a weight that signifies its relative relevance to a certain decision or study, was utilized to integrate bacterial load, coliform concentration, and proximity to pollution sources into a composite contamination risk rating (Figure 5A, B). The variables used in the model included bacterial load (0.5) and coliform concentration (0.3), both of which were recorded from each sampling site. The third variable, assigned a 0.2 weight, measured the proximity of the sampling sites to potential pollution sources. The distance to pollution sources was measured in a GIS environment. Buffer rings were generated around the pollution sources using ArcGIS Pro software, which was converted into a raster layer for use in the model. The pollution sources included public rubbish dumps, markets, and slaughterhouses, among others. All input layers were normalized to a uniform 1–5 scale before being weighed. Weights were allocated according to epidemiological significance, with bacterial burden assigned the highest weight of 0.5. The resultant contamination risk map delineates geographical variation in microbial exposure risk. An equal interval classification method was applied to generate the five levels of risk categories.

### 3.9. AMR Patterns of Pathogenic Bacteria Isolated From Food Milling Machines

**Figure 6:**
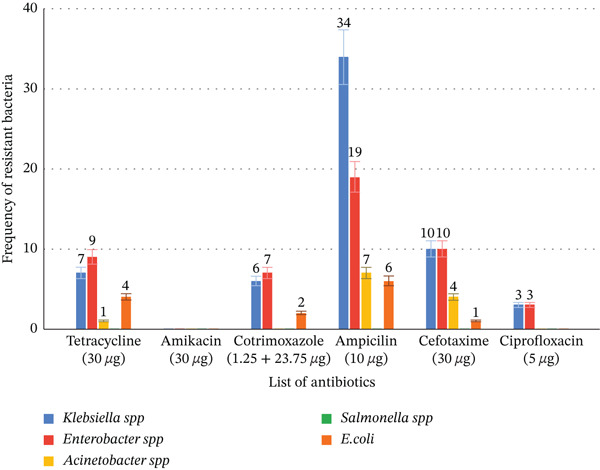
Antimicrobial resistance patterns of pathogenic isolates from food milling machines.

A summary of the AMR patterns of pathogenic bacterial isolates against six different antibiotics is shown in Figure 6. The majority of the bacterial isolates were resistant to ampicillin, with *Enterobacter* spp. being the most common, recording the highest resistance (63.33%), followed by *Klebsiella* spp. (62.96%), *E. coli* (54.54%), and *Acinetobacter* spp. (53.85%), respectively. *Salmonella* spp. isolates did not show resistance to any of the antibiotics tested. In addition, none of the isolates from any bacterial group was resistant to amikacin.

### 3.10. Demographic Information and Hygiene Practices of Food Milling Operators

Demographic information of 34 food milling machine operators in Somanya and their hygiene practices are detailed in this section. Male machine operators dominated (58.62%), while female machine operators accounted for 41.38%. A total of 86.21% were from the ages 31–61, whereas 13.79% were within the age range 20–30. One participant (3.45%) had no education, and 96.55% had at least primary, JHS, or SHS education. Most of the machine operators own the machines (93.10%), with 6.90% operating for someone else. A fair number of them have licensed their machines (48.28%), whereas 51.72% are unlicensed, with 17.24% operating two machines. Hygiene practices were encouraging, with 55.17% of the machine operators washing the machine with soap before starting work; however, none washed it with soap after each mill. A very few machine operators practiced hand hygiene (27.59%), washing their hands with soap and clean water before and after successive mills. However, a large number (72.41%) do not wash their hands with soap, only with water, before and after successive mills. No participants wore gloves or hair covers while operating the machine, leading to direct hand contact with food. Among participants who operated on fufu milling machines, 47.06% used either wood or steel to maneuver and mix food to be milled; however, none washed them with soap and water after successive milling, only with water. All participants were aware of microbial contamination resulting from unhygienic practices by the operators and from the surrounding environment, as well as the associated risks.

## 4. Discussion

The present study recorded mean bacterial and coliform loads exceeding 10^6^ CFU/mL. This is considerably higher than the standard value stipulated by the Ghana Standards Authority (GSA) (< 10^3^ CFU/g) [3]. These findings suggest significant bacterial contamination across all three milling machine types examined. This is consistent with a comparable study by Yar et al. [3] conducted in the Asante Mampong Municipality, Ghana, which similarly reported bacterial counts above GSA limits across fufu, corn, and pepper/tomato milling machines; however, the mean count in that study (1.96 × 10^5^ CFU/mL) was lower than that recorded in the present study, suggesting that contamination levels may vary by location and sanitation behavior of operators. Broader surveillance across Ghanaian food environments corroborates this pattern: A meta‐analysis of Ghanaian food safety literature identified *Enterobacter* spp., *Escherichia* spp., *Klebsiella* spp., and *Citrobacter* spp. as among the most prevalent bacterial isolates in food samples, with fufu among the most contaminated food types [14].

The present study, therefore, adds milling machine surfaces as a documented reservoir for these same organisms within the food processing chain. Fufu milling machines recorded the highest mean bacterial load among the three machine types examined. This finding is consistent with Yar et al. [3], who reported that fufu pounding machines yielded the highest number of bacterial isolates. The elevated contamination of fufu machines is possibly attributable to the sticky, paste‐like consistency of fufu, which facilitates residue adherence to grinding compartments and outlet surfaces, creating conditions that favor microbial colonization and proliferation. This physical characteristic distinguishes fufu machines from corn and pepper/tomato mills, where substrate properties offer less opportunity for residue buildup. Similarly, the Asante Mampong study reported that pepper/tomato milling machines exhibited lower microbial loads, a finding consistent with the present study, in which tomato/pepper machines also showed the lowest overall contamination levels. This observation may be attributed to the acidic pH of tomatoes and peppers, which inhibits the growth of many bacterial groups [15]. The lower bacterial load in corn mills relative to fufu mills is consistent across both studies and is partly attributable to the lower pH environment of grain‐based substrates, which restricts the colonization of pH‐sensitive microbial groups.

The similarity in microbial loads across machine types may suggest that factors beyond machine type, such as cleaning practices, environmental exposure, and repeated use without sanitation, could contribute to contamination. However, these factors were not directly measured and should be investigated in future studies. Milling machines are frequently used repeatedly without proper sanitation between uses, which may facilitate the accumulation and transfer of microorganisms regardless of machine type. Furthermore, milling environments often expose equipment to dust, insects, and other environmental contaminants that may contribute to microbial proliferation.

The coliform genera isolated from all three machine types in this study, which included *Klebsiella* spp., *E. coli*, *Acinetobacter* spp., *Salmonella* spp., and *Enterobacter* spp., are well‐documented foodborne pathogens and indicators of fecal contamination in food processing environments. Their occurrence in this study aligns closely with findings from across Ghana. A study on ready‐to‐eat (RTE) foods and vendor hand swabs in Accra identified *Enterobacter* spp. (16.8%), *Citrobacter* spp. (10.1%), *Klebsiella pneumoniae* (4.0%), and *E. coli* among the dominant isolates, noting that their presence reflected inadequate hygiene by food handlers [16]. Yeleliere et al. [14] similarly identified *Enterobacter* spp., *Escherichia* spp., *Staphylococcus* spp., and *Pseudomonas* spp. as the predominant bacteria in Ghanaian food environments across multiple regional capitals. Notably, the present study did not isolate *Shigella* spp., which Yar et al. [3] recovered from milling machines in Asante Mampong; this discrepancy may reflect genuine ecological differences between study sites or the limitations of the selective media employed. The presence of *Salmonella* spp. is particularly concerning given that *Salmonella* is a leading cause of foodborne gastroenteritis globally, responsible for an estimated 93.8 million nontyphoidal infections and 155,000 deaths annually [17], and has been repeatedly recovered from Ghanaian food and food processing environments [3, 18].

AMR profiling of the pathogenic isolates recovered from milling machine surfaces revealed a pattern consistent with broader Ghanaian AMR surveillance data. All four tested genera, *Enterobacter* spp. (63.33%), *Klebsiella* spp. (62.96%), *E. coli* (54.54%), and *Acinetobacter* spp. (53.85%) exhibited high resistance to ampicillin, while no isolate was resistant to amikacin, and *Salmonella* spp. showed no resistance to any of the six antibiotics tested. Resistance to ampicillin among these genera is not unexpected, as intrinsic beta‐lactamase activity in *Klebsiella* spp. and *Enterobacter* spp. has been documented since the 1960s [19, 20], and current surveillance confirms that ampicillin resistance now exceeds 70% in Gram‐negative clinical isolates across Ghanaian teaching hospitals [21]. The present study demonstrates that these same resistance patterns are detectable in community‐level food processing environments, suggesting that milling machine surfaces may serve as ecological reservoirs for resistant organisms beyond the clinical setting.

Of particular concern is the resistance to cefotaxime, a third‐generation cephalosporin, observed among *Enterobacter* spp. in this study. Resistance to third‐generation cephalosporins has been directly associated with higher mortality, prolonged hospitalization, and elevated treatment costs in clinical settings [22]. A recent narrative review of ESKAPEE pathogens across Ghanaian regions documented that *E. coli* and *K. pneumoniae* consistently exhibit resistance to ampicillin and third‐generation cephalosporins exceeding 70% [23]. The detection of cefotaxime resistance in food‐contact surface isolates from Somanya is, therefore, aligned with national trends and highlights the need for food environment AMR surveillance to complement clinical monitoring. When resistant organisms from milling machines contaminate food, it can lead to foodborne infections. Consumers, particularly those who are immunocompromised, elderly, or under 5 years of age, face a heightened risk of treatment failure should foodborne infection occur [24].

The AMR profiles identified in this study are consistent with those reported from other Ghanaian food environments. Dela et al. [16] isolated multidrug‐resistant *Enterobacter* spp., *K. pneumoniae*, and *E. coli* from RTE food and food vendor hand swabs in Accra, with resistance patterns comparable to those observed here. Suglo et al. [18] specifically recovered multidrug‐resistant *Salmonella* species from fufu grinding machines in Ghana, corroborating this study′s finding of *Salmonella* spp. on milling surfaces. The overall AMR burden in Ghana, estimated at 5900 attributable deaths and 25,300 associated deaths annually, and, now, AMR‐associated fatalities exceed those from malaria and HIV/AIDS, underscoring the public health urgency of including food processing environments in national surveillance frameworks [7].

From a One Health perspective, the detection of antibiotic‐resistant Gram‐negative bacteria on milling machine surfaces in Somanya represents a convergence of the human, animal, and environmental dimensions of AMR. Resistant organisms circulating in agricultural soils and irrigation water through the use of antibiotic‐treated livestock manure on food crops can colonize fresh produce and persist through the food processing chain, including milling [8, 9]. The absence of molecular characterization of resistance genes in this study means that horizontal gene transfer among milling surface isolates cannot be confirmed; however, the high prevalence of ampicillin and cefotaxime resistance across multiple genera is consistent with shared resistance determinants and warrants future genomic investigation. Integrating AMR surveillance of food processing environments into One Health strategies would strengthen Ghana′s capacity to detect and interrupt the environmental transmission of resistance.

The findings of this study highlight the urgent need for targeted interventions at policy, practice, and research levels to address microbial contamination in food milling machines. From a policy perspective, there is a need for stricter regulatory enforcement by authorities. This should include licensing requirements for milling operators and routine inspections of milling facilities to ensure compliance with food safety standards. Establishing enforceable microbial limits and penalties for noncompliance will further strengthen food safety control measures. Emphasis should be placed on improving hygiene behaviors among milling machine operators, whereas mandatory training programs on food safety and sanitation should be introduced to enhance awareness and ensure proper implementation of hygiene practices. Regular cleaning and disinfection of milling machines, especially between processing batches, should be enforced. In addition, the use of personal protective equipment such as gloves, along with proper handwashing practices, should be promoted and monitored. Routine microbial monitoring of milling environments is also essential for tracking contamination levels and ensuring continuous compliance with safety standards.

## 5. Key Findings

This study revealed several important findings regarding microbial contamination and the hygiene status of food milling machines in Somanya. First, a high microbial load was observed across all milling machine types, with values exceeding the GSA′s recommended limits, indicating significant contamination. Second, among the different machine types, fufu milling machines exhibited the highest levels of contamination. Third, hygiene practices observed at the milling sites were generally poor, with inadequate cleaning procedures and limited adherence to proper sanitation protocols. Finally, antimicrobial susceptibility testing showed a high level of resistance among bacterial isolates, particularly to commonly used antibiotics such as ampicillin, and notable resistance to cefotaxime, a third‐generation cephalosporin.

## 6. Limitations of the Study

The cross‐sectional design provides a snapshot of contamination at a single point in time and does not account for potential seasonal variation in microbial load or species composition. Surface swabs from machine inlets, grinding compartments, and outlets do not directly confirm contamination of the final milled food product; additional sampling of the milled output would be needed to establish a direct contamination pathway to consumers. Also, only Gram‐negative bacteria were targeted due to the use of MacConkey agar as the primary selective medium; Gram‐positive pathogens such as *Staphylococcus aureus* and *Listeria* spp., which are relevant foodborne pathogens, were not captured. Molecular confirmation of resistance genes was not performed; therefore, phenotypic resistance patterns cannot be attributed to specific genetic mechanisms, and the potential for horizontal gene transfer among isolates remains uncharacterized. The study did not assess the quality or source of water used by operators for machine cleaning, which is a potential additional vector for microbial contamination. Some hygiene‐related data were collected through self‐reported questionnaire responses, which may be subject to social desirability bias, potentially leading to an overestimation of compliance with hygiene practices. Finally, while MALDI‐TOF mass spectrometry is a well‐validated identification tool, it has recognized limitations in differentiating closely related species such as *E. coli* and *Shigella* spp., meaning that species‐level identification of a small subset of isolates may warrant molecular confirmation in future studies.

## 7. Conclusions and Future Studies

This study reveals that food milling machines in Somanya are heavily contaminated with various bacteria, particularly *Klebsiella* spp., posing a serious food safety risk. The bacterial load exceeded recommended standards, indicating poor hygiene and sanitation practices among operators, with fufu machines showing especially high contamination levels. Additionally, the presence of AMR, especially to ampicillin and cefotaxime, raises further public health concerns. The findings highlight inadequate management practices and low awareness of contamination risks among machine operators.

Future studies should be expanded to provide a more comprehensive picture of the Yilo Krobo municipality, including an examination of the effects of seasonal variations on contamination levels and a possible identification of Gram‐positive bacteria. Investigations must also seek to identify cross‐contamination between milling machine surfaces and the milled food. Additionally, key variables that affect hygiene, such as the type of water used for cleaning and the condition of operators′ hands, must be incorporated. Finally, future studies should consider the incorporation of molecular analysis to overcome the known weaknesses of mass spectrometry methods (MALDI‐TOF) in the accurate identification and differentiation of closely related species such as *E. coli* and *Shigella* species.

## Author Contributions

B.A.F.: writing (original draft), methodology, and data acquisition and analysis; A.K.D.: writing (original draft, review, and editing), supervision, and data analysis; S.K.L.: writing (original draft, review, and editing) and data analysis.

## Funding

No funding was received for this manuscript.

## Ethics Statement

The verbal consent of all machine operators was sought. All participants were fully informed about the study′s purpose and assured of their confidentiality. The study was fully guided by institutional protocols.

## Conflicts of Interest

The authors declare no conflicts of interest.

## Supporting information


**Supporting Information** Additional supporting information can be found online in the Supporting Information section. Table S1: (A) Questionnaire used for the study. A structured questionnaire was administered to machine operators. The questionnaire was used to gather information on hygiene practices, cleaning routines, and food safety knowledge. (B) Demographic information of milling machine operators. Demographic information was compiled based on the structured questionnaire and interview conducted. The questionnaire focused on personal hygiene, food safety practices, maintenance of milling machines, and knowledge of microbial contamination. Table S2: Bacterial species identified with MALDI‐TOF. Microbial analysis and identification using MALDI‐TOF revealed the presence of 129 bacterial species isolated from the sampled milling stations.

## Data Availability

All relevant summary data are included in the manuscript. Additional datasets, including isolate‐level data and questionnaire responses, are available from the corresponding author upon reasonable request.
