# Supplementary material for: Microbial Contamination, Hygienic Practices, and Antimicrobial Resistance Patterns of Food Milling Machines in Somanya, Ghana
Source: Int J Food Sci. 2026 Jun 17;2026:2474171. doi: 10.1155/ijfo/2474171 (PMC13273225; doi:10.1155/ijfo/2474171)
Supplement: Supplementary file 1 — Supporting Information Additional supporting information can be found online in the Supporting Information section. Table S1: (A) Questionnaire used for the study. A structured questionnaire was administered to machine operators. The questionnaire was used to gather information on hygiene practices, cleaning routines, and food safety knowledge. (B) Demographic information of milling machine operators. Demographic information was compiled based on the structured questionnaire and interview conducted. The questionnaire focused on personal hygiene, food safety practices, maintenance of milling machines, and knowledge of microbial contamination. Table S2: Bacterial species identified with MALDI‐TOF. Microbial analysis and identification using MALDI‐TOF revealed the presence of 129 bacterial species isolated from the sampled milling stations. [file IJFO-2026-2474171-s001.docx]

**SUPPLEMENTARY FILES**

**Table S1A: Questionnaire used for the study**

| 1. **Demographic and geographical data** |  |
| --- | --- |
| Id: ………………… |  |
| 1. Sex | - M - F |
| 1. Age | ___/___/___   - Unknown: Age: …… (year) |
| 1. Level of education | - *Primary* - *Junior High School* - *Senior High School* - *University* - *Uneducated* |
| 1. Residence (Location) | ……………………………………….. |
| 1. **General information** |  |
| 1. Owner of the mill | - *Yes* - *No* |
| 1. Operates mills with license | - *Yes* - *No* |
| 1. Frequency of maintaining mills | - *Once a week* - *Once a month* - *When it breaks down*   *Others specify*: …………………………… |
| 1. Do you wash the machine compartment with soap? | - *Yes* - *No*   *If yes, what is used?* ………………………………… |
| 5. Aware of microbial contamination in mills | - *Yes* - *No* |
| 6. Frequency of hand washing in a day (Hand Hygiene) | - *Once* - *Twice* - *Often* |
| 7. Wash hands with soap before operation | - *Yes* - *No* |
| 8. Wear hand gloves while milling | - *Yes* - *No* |
| 10. Is there hand contact with food while milling | - *Yes* - *No* |

**Table S1B: Demographic information of milling machine operators**

| Sampling Site | Sex | Age | Education | Machine ownership | Licensed | WMWS_or_NOT | Hand_  Hygiene | Use_gloves | Cleaning_frequency | AoMC | Use gloves |
| --- | --- | --- | --- | --- | --- | --- | --- | --- | --- | --- | --- |
| Trom (fufu) | Male | 43 | Tertiary | Yes | No | Yes | No | No | once | Yes | No |
| Ogome (corn) | Male | 41 | SHS (A-Level) | Yes | Yes | No | No | No | once | Yes | No |
| Ogome (corn) | Male | 54 | SHS (A-Level) | Yes | Yes | No | No | No | once | Yes | No |
| Ogome (fufu) | Male | 38 | SHS (A-Level) | Yes | Yes | Yes | No | No | once | Yes | No |
| Ogome (fufu) | Female | 52 | JHS | Yes | No | No | Yes | No | once | Yes | No |
| Nyawer Abokobi (corn) | Male | 27 | SHS | Yes | Yes | No | No | No | twice | Yes | No |
| Nyawer Abokobi (fufu) | Female | 42 | JHS | Yes | No | Yes | Yes | No | once | Yes | No |
| Zongo (corn) | Male | 61 | JHS | Yes | Yes | No | No | No | once | Yes | No |
| Djaba Road (fufu) | Male | 42 | Tertiary | Yes | Yes | Yes | No | No | once | Yes | No |
| Slaughter House (fufu) | Male | 37 | Tertiary | Yes | No | No | Yes | No | twice | Yes | No |
| Market (corn) | Male | 24 | SHS | Yes | Yes | Yes | No | No | once | Yes | No |
| Market (pepper/tomato) | Male | 35 | O-level | Yes | No | No | No | No | once | Yes | No |
| Salesu (corn) | Male | 48 | SHS | Yes | No | No | No | No | once | Yes | No |
| Salesu (fufu) | Female | 34 | JHS | Yes | No | Yes | Yes | No | twice | Yes | No |
| Mount Mary (corn) | Male | 50 | SHS(A-level) | Yes | No | No | No | No | twice | Yes | No |
| Mount Mary (fufu) | Female | 53 | O-level | Yes | Yes | No | Yes | No | twice | Yes | No |
| Mount Mary (Pepper/tomato) | Female | 39 | Tertiary | Yes | Yes | Yes | No | No | twice | Yes | No |
| Sawer (corn) | Male | 52 | Tertiary | Yes | Yes | Yes | No | No | once | Yes | No |
| Sawer (corn) | Male | 49 | SHS | Yes | No | No | No | No | twice | Yes | No |
| Sawer (corn) | Male | 26 | SHS | Yes | Yes | No | Yes | No | once | Yes | No |
| Sawer (pepper/tomato) | Male | 44 | No school | Yes | No | Yes | Yes | No | once | Yes | No |
| Sawer (pepper/tomato) | Female | 58 | SHS | Yes | No | No | Yes | No | twice | Yes | No |
| Sawer (fufu) | Female | 40 | SHS | No | Yes | Yes | Yes | No | twice | Yes | No |
| Sawer (fufu) | Female | 36 | SHS(A-level) | Yes | Yes | No | No | No | once | Yes | No |
| Sawer (fufu) | Female | 47 | Tertiary | Yes | No | No | Yes | No | once | Yes | No |
| Sawer (fufu) | Male | 40 | SHS (A-Level) | Yes | No | Yes | Yes | No | once | Yes | No |
| Sawer- Quarters (corn) | Male | 24 | SHS | No | No | No | No | No | twice | Yes | No |
| Roundabout (fufu) | Female | 45 | JHS | No | Yes | Yes | No | No | once | Yes | No |
| Methodist school (fufu) | Female | 31 | JHS | No | No | No | No | No | once | Yes | No |
| Methodist school (fufu) | Female | 33 | SHS (A-Level) | Yes | Yes | Yes | Yes | No | twice | Yes | No |
| SRA (fufu) | Male | 26 | SHS | Yes | No | Yes | No | No | once | Yes | No |
| SRA (corn) | Female | 35 | SHS | No | No | No | Yes | No | once | Yes | No |
| Akutunya Market (corn) | Male | 32 | SHS | Yes | No | No | No | No | once | Yes | No |
| Akutunya Market (corn) | Male | 38 | SHS (A-Level) | Yes | Yes | No | No | No | once | Yes | No |

WMWS = Wash Machine With Soap

AoMC = Aware of Microbial Contamination

**Table S2: Bacterial species identified with MALDI-TOF**

| **Sample Name** | **Sample ID** | **Organism (best match)** | **Score Value** | **Organism (second-best match)** | **Score Value** |
| --- | --- | --- | --- | --- | --- |
| [B11](https://docs.google.com/document/d/1flDt2vKlwQ_82cwowFYN6X6sJmXbqcUe/edit#heading=h.d8pwes57cpdt) (-) (C) | 2CG 10-  1  (standard) | no peaks found | [0.00](https://docs.google.com/document/d/1flDt2vKlwQ_82cwowFYN6X6sJmXbqcUe/edit#heading=h.7362t4pkur7p) | no peaks found | [0.00](https://docs.google.com/document/d/1flDt2vKlwQ_82cwowFYN6X6sJmXbqcUe/edit#heading=h.7362t4pkur7p) |
| [B12](https://docs.google.com/document/d/1flDt2vKlwQ_82cwowFYN6X6sJmXbqcUe/edit#heading=h.j4y8czlnac2k) (+) (B) | RAFO 10-2  (standard) | [*Pseudomonas putida*](https://docs.google.com/document/d/1flDt2vKlwQ_82cwowFYN6X6sJmXbqcUe/edit#heading=h.umqoett22imd) | [1.80](https://docs.google.com/document/d/1flDt2vKlwQ_82cwowFYN6X6sJmXbqcUe/edit#heading=h.7362t4pkur7p) | [*Pseudomonas monteilii*](https://docs.google.com/document/d/1flDt2vKlwQ_82cwowFYN6X6sJmXbqcUe/edit#heading=h.38242qd0ol6y) | [1.77](https://docs.google.com/document/d/1flDt2vKlwQ_82cwowFYN6X6sJmXbqcUe/edit#heading=h.7362t4pkur7p) |
| [C1](https://docs.google.com/document/d/1flDt2vKlwQ_82cwowFYN6X6sJmXbqcUe/edit#heading=h.tdcmtmryjjtn) (+) (B) | MMPI A  (standard) | [*Klebsiella pneumoniae*](https://docs.google.com/document/d/1flDt2vKlwQ_82cwowFYN6X6sJmXbqcUe/edit#heading=h.q6qq3vhxp2hf) | [1.90](https://docs.google.com/document/d/1flDt2vKlwQ_82cwowFYN6X6sJmXbqcUe/edit#heading=h.7362t4pkur7p) | [*Klebsiella pneumoniae*](https://docs.google.com/document/d/1flDt2vKlwQ_82cwowFYN6X6sJmXbqcUe/edit#heading=h.3dag4ul9oqrm) | [1.71](https://docs.google.com/document/d/1flDt2vKlwQ_82cwowFYN6X6sJmXbqcUe/edit#heading=h.7362t4pkur7p) |
| [C2](https://docs.google.com/document/d/1flDt2vKlwQ_82cwowFYN6X6sJmXbqcUe/edit#heading=h.r5pszrobpzeb)  (+) (B) | MMPI B  (standard) | *Citrobacter freundii* | [1.](https://docs.google.com/document/d/1flDt2vKlwQ_82cwowFYN6X6sJmXbqcUe/edit#heading=h.7362t4pkur7p)79 | *Citrobacter freundii* | [1.](https://docs.google.com/document/d/1flDt2vKlwQ_82cwowFYN6X6sJmXbqcUe/edit#heading=h.7362t4pkur7p)72 |
| [C3](https://docs.google.com/document/d/1flDt2vKlwQ_82cwowFYN6X6sJmXbqcUe/edit#heading=h.oh5egnx99aqg) (+) (C) | SFC A  (standard) | [*Salmonella sp*](https://docs.google.com/document/d/1flDt2vKlwQ_82cwowFYN6X6sJmXbqcUe/edit#heading=h.zb9l1o5vr9os)*p* | [1.79](https://docs.google.com/document/d/1flDt2vKlwQ_82cwowFYN6X6sJmXbqcUe/edit#heading=h.7362t4pkur7p) | *Staphylococcus aureus* | [1.72](https://docs.google.com/document/d/1flDt2vKlwQ_82cwowFYN6X6sJmXbqcUe/edit#heading=h.7362t4pkur7p) |
| [C4](https://docs.google.com/document/d/1flDt2vKlwQ_82cwowFYN6X6sJmXbqcUe/edit#heading=h.aikuc75vpsdf) (+++) (A) | SRACO 10-  2 B  (standard) | [*Escherichia coli*](https://docs.google.com/document/d/1flDt2vKlwQ_82cwowFYN6X6sJmXbqcUe/edit#heading=h.qwxxutwqd5py) | [2.11](https://docs.google.com/document/d/1flDt2vKlwQ_82cwowFYN6X6sJmXbqcUe/edit#heading=h.7362t4pkur7p) | [*Escherichia coli*](https://docs.google.com/document/d/1flDt2vKlwQ_82cwowFYN6X6sJmXbqcUe/edit#heading=h.7uv34lj781or) | [2.02](https://docs.google.com/document/d/1flDt2vKlwQ_82cwowFYN6X6sJmXbqcUe/edit#heading=h.7362t4pkur7p) |
| [C5](https://docs.google.com/document/d/1flDt2vKlwQ_82cwowFYN6X6sJmXbqcUe/edit#heading=h.bknear3gmzmq) (+++) (A) | OFO3 10- 2 A  (standard) | [*Enterobacter cloacae*](https://docs.google.com/document/d/1flDt2vKlwQ_82cwowFYN6X6sJmXbqcUe/edit#heading=h.8gksbefct7ju) | [2.16](https://docs.google.com/document/d/1flDt2vKlwQ_82cwowFYN6X6sJmXbqcUe/edit#heading=h.7362t4pkur7p) | [*Enterobacter cloacae*](https://docs.google.com/document/d/1flDt2vKlwQ_82cwowFYN6X6sJmXbqcUe/edit#heading=h.og79vjor5prn) | [2.16](https://docs.google.com/document/d/1flDt2vKlwQ_82cwowFYN6X6sJmXbqcUe/edit#heading=h.7362t4pkur7p) |
| [C6](https://docs.google.com/document/d/1flDt2vKlwQ_82cwowFYN6X6sJmXbqcUe/edit#heading=h.quz0esi1lr3y) (+++) (A) | OFO3 10- 2 B  (standard) | [*Klebsiella pneumoniae*](https://docs.google.com/document/d/1flDt2vKlwQ_82cwowFYN6X6sJmXbqcUe/edit#heading=h.q6qq3vhxp2hf) | [2.07](https://docs.google.com/document/d/1flDt2vKlwQ_82cwowFYN6X6sJmXbqcUe/edit#heading=h.7362t4pkur7p) | [*Klebsiella pneumoniae*](https://docs.google.com/document/d/1flDt2vKlwQ_82cwowFYN6X6sJmXbqcUe/edit#heading=h.srzgkoeuu3tz) | [2.03](https://docs.google.com/document/d/1flDt2vKlwQ_82cwowFYN6X6sJmXbqcUe/edit#heading=h.7362t4pkur7p) |
| [C7](https://docs.google.com/document/d/1flDt2vKlwQ_82cwowFYN6X6sJmXbqcUe/edit#heading=h.amq4kbhkdxmd) (+++) (A) | SGFG4 10-1 A  (standard) | [*Enterobacter kobei*](https://docs.google.com/document/d/1flDt2vKlwQ_82cwowFYN6X6sJmXbqcUe/edit#heading=h.6pil7oydtogv) | [2.06](https://docs.google.com/document/d/1flDt2vKlwQ_82cwowFYN6X6sJmXbqcUe/edit#heading=h.7362t4pkur7p) | [*Enterobacter asburiae*](https://docs.google.com/document/d/1flDt2vKlwQ_82cwowFYN6X6sJmXbqcUe/edit#heading=h.bpu73ci3n5u2) | [1.97](https://docs.google.com/document/d/1flDt2vKlwQ_82cwowFYN6X6sJmXbqcUe/edit#heading=h.7362t4pkur7p) |
| [C8](https://docs.google.com/document/d/1flDt2vKlwQ_82cwowFYN6X6sJmXbqcUe/edit#heading=h.66v59kl3fpe0) (+) (B) | OCO I 10-3 A  (standard) | [*Aeromonas caviae*](https://docs.google.com/document/d/1flDt2vKlwQ_82cwowFYN6X6sJmXbqcUe/edit#heading=h.8h32gzjgwp49) | [1.86](https://docs.google.com/document/d/1flDt2vKlwQ_82cwowFYN6X6sJmXbqcUe/edit#heading=h.7362t4pkur7p) | [*Aeromonas caviae*](https://docs.google.com/document/d/1flDt2vKlwQ_82cwowFYN6X6sJmXbqcUe/edit#heading=h.n9tel74p7ihk) | [1.83](https://docs.google.com/document/d/1flDt2vKlwQ_82cwowFYN6X6sJmXbqcUe/edit#heading=h.7362t4pkur7p) |
| [C9](https://docs.google.com/document/d/1flDt2vKlwQ_82cwowFYN6X6sJmXbqcUe/edit#heading=h.gt9alkeh86fh) (+++) (A) | SRACO 10-2 A  (standard) | [*Escherichia coli*](https://docs.google.com/document/d/1flDt2vKlwQ_82cwowFYN6X6sJmXbqcUe/edit#heading=h.q70dpxy67t6h) | [2.27](https://docs.google.com/document/d/1flDt2vKlwQ_82cwowFYN6X6sJmXbqcUe/edit#heading=h.7362t4pkur7p) | [*Escherichia coli*](https://docs.google.com/document/d/1flDt2vKlwQ_82cwowFYN6X6sJmXbqcUe/edit#heading=h.7uv34lj781or) | [2.21](https://docs.google.com/document/d/1flDt2vKlwQ_82cwowFYN6X6sJmXbqcUe/edit#heading=h.7362t4pkur7p) |
| [C10](https://docs.google.com/document/d/1flDt2vKlwQ_82cwowFYN6X6sJmXbqcUe/edit#heading=h.vhpz3dciab0b) (+++) (A) | OFO I A  (standard) | *Klebsiella pneumoniae* | 2.03 | *Klebsiella pneumoniae* | 2.00 |
| [C11](https://docs.google.com/document/d/1flDt2vKlwQ_82cwowFYN6X6sJmXbqcUe/edit#heading=h.t0flp2epr66b) (-) (C) | OFO I B  (standard) | no peaks found | [0.00](https://docs.google.com/document/d/1flDt2vKlwQ_82cwowFYN6X6sJmXbqcUe/edit#heading=h.7362t4pkur7p) | no peaks found | [0.00](https://docs.google.com/document/d/1flDt2vKlwQ_82cwowFYN6X6sJmXbqcUe/edit#heading=h.7362t4pkur7p) |
| [C12](https://docs.google.com/document/d/1flDt2vKlwQ_82cwowFYN6X6sJmXbqcUe/edit#heading=h.p3skj1oia5zb) (-) (C) | OFO I C  (standard) | no peaks found | [0.00](https://docs.google.com/document/d/1flDt2vKlwQ_82cwowFYN6X6sJmXbqcUe/edit#heading=h.7362t4pkur7p) | no peaks found | [0.00](https://docs.google.com/document/d/1flDt2vKlwQ_82cwowFYN6X6sJmXbqcUe/edit#heading=h.7362t4pkur7p) |
| [D1](https://docs.google.com/document/d/1flDt2vKlwQ_82cwowFYN6X6sJmXbqcUe/edit#heading=h.pfrbmym7nwxf) (+++) (A) | OFGI A  (standard) | *[Enterobacter roggenkampii](https://docs.google.com/document/d/1flDt2vKlwQ_82cwowFYN6X6sJmXbqcUe/edit#heading=h.mys7i7b1ymle)* | [2.16](https://docs.google.com/document/d/1flDt2vKlwQ_82cwowFYN6X6sJmXbqcUe/edit#heading=h.7362t4pkur7p) | [*Enterobacter roggenkampii*](https://docs.google.com/document/d/1flDt2vKlwQ_82cwowFYN6X6sJmXbqcUe/edit#heading=h.duu06us016k5) | [2.15](https://docs.google.com/document/d/1flDt2vKlwQ_82cwowFYN6X6sJmXbqcUe/edit#heading=h.7362t4pkur7p) |
| [D2](https://docs.google.com/document/d/1flDt2vKlwQ_82cwowFYN6X6sJmXbqcUe/edit#heading=h.7ef1ttp2s63r) (+++) (A) | OFGI B  (standard) | [*Klebsiella variicola*](https://docs.google.com/document/d/1flDt2vKlwQ_82cwowFYN6X6sJmXbqcUe/edit#heading=h.g57ok72ztt7z) | [2.18](https://docs.google.com/document/d/1flDt2vKlwQ_82cwowFYN6X6sJmXbqcUe/edit#heading=h.7362t4pkur7p) | [*Klebsiella variicola*](https://docs.google.com/document/d/1flDt2vKlwQ_82cwowFYN6X6sJmXbqcUe/edit#heading=h.g3cxb5kr6azs) | [2.14](https://docs.google.com/document/d/1flDt2vKlwQ_82cwowFYN6X6sJmXbqcUe/edit#heading=h.7362t4pkur7p) |
| [D3](https://docs.google.com/document/d/1flDt2vKlwQ_82cwowFYN6X6sJmXbqcUe/edit#heading=h.4fpv0b8hbz1w) (+++) (A) | OFGI C  (standard) | [*Klebsiella pneumoniae*](https://docs.google.com/document/d/1flDt2vKlwQ_82cwowFYN6X6sJmXbqcUe/edit#heading=h.q6qq3vhxp2hf) | [2.27](https://docs.google.com/document/d/1flDt2vKlwQ_82cwowFYN6X6sJmXbqcUe/edit#heading=h.7362t4pkur7p) | [*Klebsiella pneumoniae*](https://docs.google.com/document/d/1flDt2vKlwQ_82cwowFYN6X6sJmXbqcUe/edit#heading=h.srzgkoeuu3tz) | [2.07](https://docs.google.com/document/d/1flDt2vKlwQ_82cwowFYN6X6sJmXbqcUe/edit#heading=h.7362t4pkur7p) |
| [D4](https://docs.google.com/document/d/1flDt2vKlwQ_82cwowFYN6X6sJmXbqcUe/edit#heading=h.htnrncfgcf35) (+++) (A) | OCG2 C  (standard) | [*Enterobacter roggenkampii*](https://docs.google.com/document/d/1flDt2vKlwQ_82cwowFYN6X6sJmXbqcUe/edit#heading=h.f9ttjghdjed) | [2.13](https://docs.google.com/document/d/1flDt2vKlwQ_82cwowFYN6X6sJmXbqcUe/edit#heading=h.7362t4pkur7p) | [*Enterobacter roggenkampii*](https://docs.google.com/document/d/1flDt2vKlwQ_82cwowFYN6X6sJmXbqcUe/edit#heading=h.1xiv9h8nxmfz) | [2.08](https://docs.google.com/document/d/1flDt2vKlwQ_82cwowFYN6X6sJmXbqcUe/edit#heading=h.7362t4pkur7p) |
| [D5](https://docs.google.com/document/d/1flDt2vKlwQ_82cwowFYN6X6sJmXbqcUe/edit#heading=h.nzqulocbyaer)  (-) (C) | SACG B  (standard) | no peaks found | [0.00](https://docs.google.com/document/d/1flDt2vKlwQ_82cwowFYN6X6sJmXbqcUe/edit#heading=h.7362t4pkur7p) | no peaks found | [0.00](https://docs.google.com/document/d/1flDt2vKlwQ_82cwowFYN6X6sJmXbqcUe/edit#heading=h.7362t4pkur7p) |
| [D6](https://docs.google.com/document/d/1flDt2vKlwQ_82cwowFYN6X6sJmXbqcUe/edit#heading=h.71cuevw6n0tg) (+++) (A) | SACG C  (standard) | [*Klebsiella pneumoniae*](https://docs.google.com/document/d/1flDt2vKlwQ_82cwowFYN6X6sJmXbqcUe/edit#heading=h.q6qq3vhxp2hf) | [2.37](https://docs.google.com/document/d/1flDt2vKlwQ_82cwowFYN6X6sJmXbqcUe/edit#heading=h.7362t4pkur7p) | [*Klebsiella pneumoniae*](https://docs.google.com/document/d/1flDt2vKlwQ_82cwowFYN6X6sJmXbqcUe/edit#heading=h.srzgkoeuu3tz) | [2.31](https://docs.google.com/document/d/1flDt2vKlwQ_82cwowFYN6X6sJmXbqcUe/edit#heading=h.7362t4pkur7p) |
| [D7](https://docs.google.com/document/d/1flDt2vKlwQ_82cwowFYN6X6sJmXbqcUe/edit#heading=h.y3459cwokf3j) (+++) (B) | SACO A  (standard) | [*Klebsiella pneumoniae*](https://docs.google.com/document/d/1flDt2vKlwQ_82cwowFYN6X6sJmXbqcUe/edit#heading=h.q6qq3vhxp2hf) | [2.05](https://docs.google.com/document/d/1flDt2vKlwQ_82cwowFYN6X6sJmXbqcUe/edit#heading=h.7362t4pkur7p) | [*Klebsiella variicola*](https://docs.google.com/document/d/1flDt2vKlwQ_82cwowFYN6X6sJmXbqcUe/edit#heading=h.g3cxb5kr6azs) | [2.01](https://docs.google.com/document/d/1flDt2vKlwQ_82cwowFYN6X6sJmXbqcUe/edit#heading=h.7362t4pkur7p) |
| [D8](https://docs.google.com/document/d/1flDt2vKlwQ_82cwowFYN6X6sJmXbqcUe/edit#heading=h.c8q18z913imn) (+++) (A) | SACO C  (standard) | [*Enterobacter bugandensis*](https://docs.google.com/document/d/1flDt2vKlwQ_82cwowFYN6X6sJmXbqcUe/edit#heading=h.dr1w9xglgtey) | [2.29](https://docs.google.com/document/d/1flDt2vKlwQ_82cwowFYN6X6sJmXbqcUe/edit#heading=h.7362t4pkur7p) | [*Enterobacter bugandensis*](https://docs.google.com/document/d/1flDt2vKlwQ_82cwowFYN6X6sJmXbqcUe/edit#heading=h.kcjigornldx0) | [2.24](https://docs.google.com/document/d/1flDt2vKlwQ_82cwowFYN6X6sJmXbqcUe/edit#heading=h.7362t4pkur7p) |
| [D9](https://docs.google.com/document/d/1flDt2vKlwQ_82cwowFYN6X6sJmXbqcUe/edit#heading=h.j76vecfbpmcz) (+++) (A) | SACG A  (standard) | [*Cronobacter* sp](https://docs.google.com/document/d/1flDt2vKlwQ_82cwowFYN6X6sJmXbqcUe/edit#heading=h.suh5hpmmxej4). | [2.24](https://docs.google.com/document/d/1flDt2vKlwQ_82cwowFYN6X6sJmXbqcUe/edit#heading=h.7362t4pkur7p) | [*Cronobacter* sp](https://docs.google.com/document/d/1flDt2vKlwQ_82cwowFYN6X6sJmXbqcUe/edit#heading=h.z7rinx6488bt). | [2.24](https://docs.google.com/document/d/1flDt2vKlwQ_82cwowFYN6X6sJmXbqcUe/edit#heading=h.7362t4pkur7p) |
| [D10](https://docs.google.com/document/d/1flDt2vKlwQ_82cwowFYN6X6sJmXbqcUe/edit#heading=h.zh7lpvwicohd) (+) (B) | OC I A  (standard) | *Aeromonas caviae* | 1.84 | *Aeromonas caviae* | 1.76 |
| [D11](https://docs.google.com/document/d/1flDt2vKlwQ_82cwowFYN6X6sJmXbqcUe/edit#heading=h.w4sl7f8io2tt) (+) (B) | ACO 10-2 B *  (standard) | *Enterobacter hormaechei* | [1.](https://docs.google.com/document/d/1flDt2vKlwQ_82cwowFYN6X6sJmXbqcUe/edit#heading=h.7362t4pkur7p)77 | *Enterobacter hormaechei* | [1.](https://docs.google.com/document/d/1flDt2vKlwQ_82cwowFYN6X6sJmXbqcUe/edit#heading=h.7362t4pkur7p)73 |
| [D12](https://docs.google.com/document/d/1flDt2vKlwQ_82cwowFYN6X6sJmXbqcUe/edit#heading=h.qymj3aquhc4e) (+++) (A) | OCG 2 A  (standard) | [*Acinetobacter baumannii*](https://docs.google.com/document/d/1flDt2vKlwQ_82cwowFYN6X6sJmXbqcUe/edit#heading=h.xp5azwfixjx) | [2.24](https://docs.google.com/document/d/1flDt2vKlwQ_82cwowFYN6X6sJmXbqcUe/edit#heading=h.7362t4pkur7p) | [*Acinetobacter baumannii*](https://docs.google.com/document/d/1flDt2vKlwQ_82cwowFYN6X6sJmXbqcUe/edit#heading=h.wbvfjlta26fg) | [2.18](https://docs.google.com/document/d/1flDt2vKlwQ_82cwowFYN6X6sJmXbqcUe/edit#heading=h.7362t4pkur7p) |
| [E1](https://docs.google.com/document/d/1flDt2vKlwQ_82cwowFYN6X6sJmXbqcUe/edit#heading=h.h6sxxm8kt20h)  (-) (C) | OFI3 A  (standard) | no peaks found | [0.00](https://docs.google.com/document/d/1flDt2vKlwQ_82cwowFYN6X6sJmXbqcUe/edit#heading=h.7362t4pkur7p) | no peaks found | [0.00](https://docs.google.com/document/d/1flDt2vKlwQ_82cwowFYN6X6sJmXbqcUe/edit#heading=h.7362t4pkur7p) |
| [E2](https://docs.google.com/document/d/1flDt2vKlwQ_82cwowFYN6X6sJmXbqcUe/edit#heading=h.f08wjnean8v) (+++) (A) | OFI3 B  (standard) | [*Klebsiella pneumoniae*](https://docs.google.com/document/d/1flDt2vKlwQ_82cwowFYN6X6sJmXbqcUe/edit#heading=h.q6qq3vhxp2hf) | [2.04](https://docs.google.com/document/d/1flDt2vKlwQ_82cwowFYN6X6sJmXbqcUe/edit#heading=h.7362t4pkur7p) | [*Klebsiella pneumoniae*](https://docs.google.com/document/d/1flDt2vKlwQ_82cwowFYN6X6sJmXbqcUe/edit#heading=h.3dag4ul9oqrm) | [1.93](https://docs.google.com/document/d/1flDt2vKlwQ_82cwowFYN6X6sJmXbqcUe/edit#heading=h.7362t4pkur7p) |
| [E3](https://docs.google.com/document/d/1flDt2vKlwQ_82cwowFYN6X6sJmXbqcUe/edit#heading=h.2tn0owovu3ji) (+++) (A) | ACO 10-2 B  (standard) | [*Klebsiella pneumoniae*](https://docs.google.com/document/d/1flDt2vKlwQ_82cwowFYN6X6sJmXbqcUe/edit#heading=h.l8o9l3joithh) | [2.03](https://docs.google.com/document/d/1flDt2vKlwQ_82cwowFYN6X6sJmXbqcUe/edit#heading=h.7362t4pkur7p) | [*Klebsiella pneumoniae*](https://docs.google.com/document/d/1flDt2vKlwQ_82cwowFYN6X6sJmXbqcUe/edit#heading=h.q6qq3vhxp2hf) | [2.00](https://docs.google.com/document/d/1flDt2vKlwQ_82cwowFYN6X6sJmXbqcUe/edit#heading=h.7362t4pkur7p) |
| [E4](https://docs.google.com/document/d/1flDt2vKlwQ_82cwowFYN6X6sJmXbqcUe/edit#heading=h.v19gjbc9e2ba)  (-) (C) | ACO 10-2 C  (standard) | No Organism Identification Possible | [1.55](https://docs.google.com/document/d/1flDt2vKlwQ_82cwowFYN6X6sJmXbqcUe/edit#heading=h.7362t4pkur7p) | No Organism Identification Possible | [1.36](https://docs.google.com/document/d/1flDt2vKlwQ_82cwowFYN6X6sJmXbqcUe/edit#heading=h.7362t4pkur7p) |
| [E5](https://docs.google.com/document/d/1flDt2vKlwQ_82cwowFYN6X6sJmXbqcUe/edit#heading=h.h1849cr92ih4)  (+) (B) | QCO 10-1 B  (standard) | *Citrobacter freundii* | [1.](https://docs.google.com/document/d/1flDt2vKlwQ_82cwowFYN6X6sJmXbqcUe/edit#heading=h.7362t4pkur7p)89 | *Citrobacter freundii* | [1.](https://docs.google.com/document/d/1flDt2vKlwQ_82cwowFYN6X6sJmXbqcUe/edit#heading=h.7362t4pkur7p)76 |
| [E6](https://docs.google.com/document/d/1flDt2vKlwQ_82cwowFYN6X6sJmXbqcUe/edit#heading=h.uaop45osjb0) (+++) (A) | RAFI 10-1  (standard) | [*Acinetobacter baumannii*](https://docs.google.com/document/d/1flDt2vKlwQ_82cwowFYN6X6sJmXbqcUe/edit#heading=h.jid6q6fuuqop) | [2.08](https://docs.google.com/document/d/1flDt2vKlwQ_82cwowFYN6X6sJmXbqcUe/edit#heading=h.7362t4pkur7p) | [*Acinetobacter baumannii*](https://docs.google.com/document/d/1flDt2vKlwQ_82cwowFYN6X6sJmXbqcUe/edit#heading=h.lqcf5fa3xof) | [2.03](https://docs.google.com/document/d/1flDt2vKlwQ_82cwowFYN6X6sJmXbqcUe/edit#heading=h.7362t4pkur7p) |
| [E7](https://docs.google.com/document/d/1flDt2vKlwQ_82cwowFYN6X6sJmXbqcUe/edit#heading=h.mbfrq3pxu47z)  (+++) (A) | ACI2 10-3 A  (standard) | *Klebsiella pneumoniae* | 2.54 | *Klebsiella pneumoniae* | 2.22 |
| [E8](https://docs.google.com/document/d/1flDt2vKlwQ_82cwowFYN6X6sJmXbqcUe/edit#heading=h.ve9qyeeldajn) (+++) (A) | SAF04 10-3 A  (standard) | [*Klebsiella pneumoniae*](https://docs.google.com/document/d/1flDt2vKlwQ_82cwowFYN6X6sJmXbqcUe/edit#heading=h.q6qq3vhxp2hf) | [2.14](https://docs.google.com/document/d/1flDt2vKlwQ_82cwowFYN6X6sJmXbqcUe/edit#heading=h.7362t4pkur7p) | [*Klebsiella pneumoniae*](https://docs.google.com/document/d/1flDt2vKlwQ_82cwowFYN6X6sJmXbqcUe/edit#heading=h.srzgkoeuu3tz) | [2.07](https://docs.google.com/document/d/1flDt2vKlwQ_82cwowFYN6X6sJmXbqcUe/edit#heading=h.7362t4pkur7p) |
| [E9](https://docs.google.com/document/d/1flDt2vKlwQ_82cwowFYN6X6sJmXbqcUe/edit#heading=h.7wt87kk1v40l) (+++) (A) | QCI A  (standard) | [*Acinetobacter baumannii*](https://docs.google.com/document/d/1flDt2vKlwQ_82cwowFYN6X6sJmXbqcUe/edit#heading=h.jid6q6fuuqop) | [2.11](https://docs.google.com/document/d/1flDt2vKlwQ_82cwowFYN6X6sJmXbqcUe/edit#heading=h.7362t4pkur7p) | [*Acinetobacter baumannii*](https://docs.google.com/document/d/1flDt2vKlwQ_82cwowFYN6X6sJmXbqcUe/edit#heading=h.xp5azwfixjx) | [2.11](https://docs.google.com/document/d/1flDt2vKlwQ_82cwowFYN6X6sJmXbqcUe/edit#heading=h.7362t4pkur7p) |
| [E10](https://docs.google.com/document/d/1flDt2vKlwQ_82cwowFYN6X6sJmXbqcUe/edit#heading=h.gng1sdr70h71) (-) (C) | ACG 2  10-4 B  (standard) | No Organism Identification Possible | [1.57](https://docs.google.com/document/d/1flDt2vKlwQ_82cwowFYN6X6sJmXbqcUe/edit#heading=h.7362t4pkur7p) | No Organism Identification Possible | [1.44](https://docs.google.com/document/d/1flDt2vKlwQ_82cwowFYN6X6sJmXbqcUe/edit#heading=h.7362t4pkur7p) |
| [E11](https://docs.google.com/document/d/1flDt2vKlwQ_82cwowFYN6X6sJmXbqcUe/edit#heading=h.s1owhu3653bb) (+++) (A) | ACG 2 10-4 A  (standard) | [*Klebsiella pneumoniae*](https://docs.google.com/document/d/1flDt2vKlwQ_82cwowFYN6X6sJmXbqcUe/edit#heading=h.q6qq3vhxp2hf) | [2.31](https://docs.google.com/document/d/1flDt2vKlwQ_82cwowFYN6X6sJmXbqcUe/edit#heading=h.7362t4pkur7p) | [*Klebsiella pneumoniae*](https://docs.google.com/document/d/1flDt2vKlwQ_82cwowFYN6X6sJmXbqcUe/edit#heading=h.srzgkoeuu3tz) | [2.29](https://docs.google.com/document/d/1flDt2vKlwQ_82cwowFYN6X6sJmXbqcUe/edit#heading=h.7362t4pkur7p) |
| [E12](https://docs.google.com/document/d/1flDt2vKlwQ_82cwowFYN6X6sJmXbqcUe/edit#heading=h.y765xukaroao) (+++) (A) | NGFI 10-1 A  (standard) | [*Klebsiella pneumoniae*](https://docs.google.com/document/d/1flDt2vKlwQ_82cwowFYN6X6sJmXbqcUe/edit#heading=h.q6qq3vhxp2hf) | [2.07](https://docs.google.com/document/d/1flDt2vKlwQ_82cwowFYN6X6sJmXbqcUe/edit#heading=h.7362t4pkur7p) | [*Klebsiella pneumoniae*](https://docs.google.com/document/d/1flDt2vKlwQ_82cwowFYN6X6sJmXbqcUe/edit#heading=h.srzgkoeuu3tz) | [1.90](https://docs.google.com/document/d/1flDt2vKlwQ_82cwowFYN6X6sJmXbqcUe/edit#heading=h.7362t4pkur7p) |
| [F1](https://docs.google.com/document/d/1flDt2vKlwQ_82cwowFYN6X6sJmXbqcUe/edit#heading=h.1w1a73ov1ay4) (+++) (A) | NGFI 10-1 B  (standard) | [*Klebsiella pneumoniae*](https://docs.google.com/document/d/1flDt2vKlwQ_82cwowFYN6X6sJmXbqcUe/edit#heading=h.q6qq3vhxp2hf) | [2.06](https://docs.google.com/document/d/1flDt2vKlwQ_82cwowFYN6X6sJmXbqcUe/edit#heading=h.7362t4pkur7p) | [*Klebsiella pneumoniae*](https://docs.google.com/document/d/1flDt2vKlwQ_82cwowFYN6X6sJmXbqcUe/edit#heading=h.srzgkoeuu3tz) | [1.93](https://docs.google.com/document/d/1flDt2vKlwQ_82cwowFYN6X6sJmXbqcUe/edit#heading=h.7362t4pkur7p) |
| [F2](https://docs.google.com/document/d/1flDt2vKlwQ_82cwowFYN6X6sJmXbqcUe/edit#heading=h.yahfm3n0rof7) (+++) (A) | NAFO 10-1 A  (standard) | [*Klebsiella variicola*](https://docs.google.com/document/d/1flDt2vKlwQ_82cwowFYN6X6sJmXbqcUe/edit#heading=h.9krxsw4zcqk) | [2.07](https://docs.google.com/document/d/1flDt2vKlwQ_82cwowFYN6X6sJmXbqcUe/edit#heading=h.7362t4pkur7p) | [*Klebsiella variicola*](https://docs.google.com/document/d/1flDt2vKlwQ_82cwowFYN6X6sJmXbqcUe/edit#heading=h.g57ok72ztt7z) | [2.07](https://docs.google.com/document/d/1flDt2vKlwQ_82cwowFYN6X6sJmXbqcUe/edit#heading=h.7362t4pkur7p) |
| [F3](https://docs.google.com/document/d/1flDt2vKlwQ_82cwowFYN6X6sJmXbqcUe/edit#heading=h.2dt28n9qy2oi)  (-) (C) | MeFG2 A  (standard) | no peaks found | [0.00](https://docs.google.com/document/d/1flDt2vKlwQ_82cwowFYN6X6sJmXbqcUe/edit#heading=h.7362t4pkur7p) | no peaks found | [0.00](https://docs.google.com/document/d/1flDt2vKlwQ_82cwowFYN6X6sJmXbqcUe/edit#heading=h.7362t4pkur7p) |
| [F4](https://docs.google.com/document/d/1flDt2vKlwQ_82cwowFYN6X6sJmXbqcUe/edit#heading=h.thyvp4gyz3zx) (+) (B) | MeFG2 C  (standard) | [*Acinetobacter baumannii*](https://docs.google.com/document/d/1flDt2vKlwQ_82cwowFYN6X6sJmXbqcUe/edit#heading=h.jid6q6fuuqop) | [1.85](https://docs.google.com/document/d/1flDt2vKlwQ_82cwowFYN6X6sJmXbqcUe/edit#heading=h.7362t4pkur7p) | [*Acinetobacter baumannii*](https://docs.google.com/document/d/1flDt2vKlwQ_82cwowFYN6X6sJmXbqcUe/edit#heading=h.lqcf5fa3xof) | [1.80](https://docs.google.com/document/d/1flDt2vKlwQ_82cwowFYN6X6sJmXbqcUe/edit#heading=h.7362t4pkur7p) |
| [F5](https://docs.google.com/document/d/1flDt2vKlwQ_82cwowFYN6X6sJmXbqcUe/edit#heading=h.mvpibvu9j6o8)  (-) (C) | SaFG1 A  (standard) | No Organism Identification Possible | [1.52](https://docs.google.com/document/d/1flDt2vKlwQ_82cwowFYN6X6sJmXbqcUe/edit#heading=h.7362t4pkur7p) | No Organism Identification Possible | [1.51](https://docs.google.com/document/d/1flDt2vKlwQ_82cwowFYN6X6sJmXbqcUe/edit#heading=h.7362t4pkur7p) |
| [F6](https://docs.google.com/document/d/1flDt2vKlwQ_82cwowFYN6X6sJmXbqcUe/edit#heading=h.3ljj3mqe10fo) (+) (B) | SaFG1 B  (standard) | [*Pseudomonas monteilii*](https://docs.google.com/document/d/1flDt2vKlwQ_82cwowFYN6X6sJmXbqcUe/edit#heading=h.xpxmlpa6b4nv) | [1.83](https://docs.google.com/document/d/1flDt2vKlwQ_82cwowFYN6X6sJmXbqcUe/edit#heading=h.7362t4pkur7p) | No Organism Identification Possible | [1.68](https://docs.google.com/document/d/1flDt2vKlwQ_82cwowFYN6X6sJmXbqcUe/edit#heading=h.7362t4pkur7p) |
| [F7](https://docs.google.com/document/d/1flDt2vKlwQ_82cwowFYN6X6sJmXbqcUe/edit#heading=h.5d6hrvabm5nd) (+++) (A) | SFI A  (standard) | [*Klebsiella pneumoniae*](https://docs.google.com/document/d/1flDt2vKlwQ_82cwowFYN6X6sJmXbqcUe/edit#heading=h.q6qq3vhxp2hf) | [2.34](https://docs.google.com/document/d/1flDt2vKlwQ_82cwowFYN6X6sJmXbqcUe/edit#heading=h.7362t4pkur7p) | [*Klebsiella pneumoniae*](https://docs.google.com/document/d/1flDt2vKlwQ_82cwowFYN6X6sJmXbqcUe/edit#heading=h.srzgkoeuu3tz) | [2.24](https://docs.google.com/document/d/1flDt2vKlwQ_82cwowFYN6X6sJmXbqcUe/edit#heading=h.7362t4pkur7p) |
| [F8](https://docs.google.com/document/d/1flDt2vKlwQ_82cwowFYN6X6sJmXbqcUe/edit#heading=h.1eeh2zpm5os4) (+++) (A) | SaPTO C  (standard) | [*Enterobacter cloacae*](https://docs.google.com/document/d/1flDt2vKlwQ_82cwowFYN6X6sJmXbqcUe/edit#heading=h.8gksbefct7ju) | [2.24](https://docs.google.com/document/d/1flDt2vKlwQ_82cwowFYN6X6sJmXbqcUe/edit#heading=h.7362t4pkur7p) | [*Enterobacter cloacae*](https://docs.google.com/document/d/1flDt2vKlwQ_82cwowFYN6X6sJmXbqcUe/edit#heading=h.og79vjor5prn) | [2.20](https://docs.google.com/document/d/1flDt2vKlwQ_82cwowFYN6X6sJmXbqcUe/edit#heading=h.7362t4pkur7p) |
| [F9](https://docs.google.com/document/d/1flDt2vKlwQ_82cwowFYN6X6sJmXbqcUe/edit#heading=h.fycuhkuxwl30) (+++) (A) | SaPTO B  (standard) | [*Citrobacter freundii*](https://docs.google.com/document/d/1flDt2vKlwQ_82cwowFYN6X6sJmXbqcUe/edit#heading=h.f5dnlsra9wmp) | [2.17](https://docs.google.com/document/d/1flDt2vKlwQ_82cwowFYN6X6sJmXbqcUe/edit#heading=h.7362t4pkur7p) | [*Citrobacter freundii*](https://docs.google.com/document/d/1flDt2vKlwQ_82cwowFYN6X6sJmXbqcUe/edit#heading=h.t5w1pj7vus4c) | [2.16](https://docs.google.com/document/d/1flDt2vKlwQ_82cwowFYN6X6sJmXbqcUe/edit#heading=h.7362t4pkur7p) |
| [F10](https://docs.google.com/document/d/1flDt2vKlwQ_82cwowFYN6X6sJmXbqcUe/edit#heading=h.9pn0qcumt0vy) (+++) (A) | SaPTO A  (standard) | [*Klebsiella pneumoniae*](https://docs.google.com/document/d/1flDt2vKlwQ_82cwowFYN6X6sJmXbqcUe/edit#heading=h.l8o9l3joithh) | [2.26](https://docs.google.com/document/d/1flDt2vKlwQ_82cwowFYN6X6sJmXbqcUe/edit#heading=h.7362t4pkur7p) | [*Klebsiella pneumoniae*](https://docs.google.com/document/d/1flDt2vKlwQ_82cwowFYN6X6sJmXbqcUe/edit#heading=h.srzgkoeuu3tz) | [2.22](https://docs.google.com/document/d/1flDt2vKlwQ_82cwowFYN6X6sJmXbqcUe/edit#heading=h.7362t4pkur7p) |
| [F11](https://docs.google.com/document/d/1flDt2vKlwQ_82cwowFYN6X6sJmXbqcUe/edit#heading=h.abrxvmidteq) (+++) (A) | SaPIG 10-1  (standard) | [*Klebsiella pneumoniae*](https://docs.google.com/document/d/1flDt2vKlwQ_82cwowFYN6X6sJmXbqcUe/edit#heading=h.q6qq3vhxp2hf) | [2.21](https://docs.google.com/document/d/1flDt2vKlwQ_82cwowFYN6X6sJmXbqcUe/edit#heading=h.7362t4pkur7p) | [*Klebsiella pneumoniae*](https://docs.google.com/document/d/1flDt2vKlwQ_82cwowFYN6X6sJmXbqcUe/edit#heading=h.odorjocrwyka) | [2.04](https://docs.google.com/document/d/1flDt2vKlwQ_82cwowFYN6X6sJmXbqcUe/edit#heading=h.7362t4pkur7p) |
| [F12](https://docs.google.com/document/d/1flDt2vKlwQ_82cwowFYN6X6sJmXbqcUe/edit#heading=h.gtoydpipav3k) (+) (B) | SACO 10-2 C  (standard) | [*Cronobacter* sp](https://docs.google.com/document/d/1flDt2vKlwQ_82cwowFYN6X6sJmXbqcUe/edit#heading=h.ejpgdxfn3w03). | [1.79](https://docs.google.com/document/d/1flDt2vKlwQ_82cwowFYN6X6sJmXbqcUe/edit#heading=h.7362t4pkur7p) | No Organism Identification Possible | [1.65](https://docs.google.com/document/d/1flDt2vKlwQ_82cwowFYN6X6sJmXbqcUe/edit#heading=h.7362t4pkur7p) |
| [G1](https://docs.google.com/document/d/1flDt2vKlwQ_82cwowFYN6X6sJmXbqcUe/edit#heading=h.z2zvjjuy4gvg)  (-) (C) | SACO2 10-2 A  (standard) | no peaks found | [0.00](https://docs.google.com/document/d/1flDt2vKlwQ_82cwowFYN6X6sJmXbqcUe/edit#heading=h.7362t4pkur7p) | no peaks found | [0.00](https://docs.google.com/document/d/1flDt2vKlwQ_82cwowFYN6X6sJmXbqcUe/edit#heading=h.7362t4pkur7p) |
| [G2](https://docs.google.com/document/d/1flDt2vKlwQ_82cwowFYN6X6sJmXbqcUe/edit#heading=h.v4puykp1grgh) (+++) (A) | DRFI 10-2  (standard) | [*Klebsiella pneumoniae*](https://docs.google.com/document/d/1flDt2vKlwQ_82cwowFYN6X6sJmXbqcUe/edit#heading=h.q6qq3vhxp2hf) | [2.19](https://docs.google.com/document/d/1flDt2vKlwQ_82cwowFYN6X6sJmXbqcUe/edit#heading=h.7362t4pkur7p) | [*Klebsiella pneumoniae*](https://docs.google.com/document/d/1flDt2vKlwQ_82cwowFYN6X6sJmXbqcUe/edit#heading=h.srzgkoeuu3tz) | [2.11](https://docs.google.com/document/d/1flDt2vKlwQ_82cwowFYN6X6sJmXbqcUe/edit#heading=h.7362t4pkur7p) |
| [G3](https://docs.google.com/document/d/1flDt2vKlwQ_82cwowFYN6X6sJmXbqcUe/edit#heading=h.e3hx11f52pq3) (+) (B) | SAFO (2) 10 -  4 A  (standard) | [*Pseudomonas monteilii*](https://docs.google.com/document/d/1flDt2vKlwQ_82cwowFYN6X6sJmXbqcUe/edit#heading=h.38242qd0ol6y) | [1.92](https://docs.google.com/document/d/1flDt2vKlwQ_82cwowFYN6X6sJmXbqcUe/edit#heading=h.7362t4pkur7p) | [*Pseudomonas monteilii*](https://docs.google.com/document/d/1flDt2vKlwQ_82cwowFYN6X6sJmXbqcUe/edit#heading=h.o37bqul9n254) | [1.78](https://docs.google.com/document/d/1flDt2vKlwQ_82cwowFYN6X6sJmXbqcUe/edit#heading=h.7362t4pkur7p) |
| [G4](https://docs.google.com/document/d/1flDt2vKlwQ_82cwowFYN6X6sJmXbqcUe/edit#heading=h.f62cokkpl5fb) (+) (B) | SAFO (2) 10 -  4 B  (standard) | [*Klebsiella pneumoniae*](https://docs.google.com/document/d/1flDt2vKlwQ_82cwowFYN6X6sJmXbqcUe/edit#heading=h.q6qq3vhxp2hf) | [1.79](https://docs.google.com/document/d/1flDt2vKlwQ_82cwowFYN6X6sJmXbqcUe/edit#heading=h.7362t4pkur7p) | No Organism Identification Possible | [1.64](https://docs.google.com/document/d/1flDt2vKlwQ_82cwowFYN6X6sJmXbqcUe/edit#heading=h.7362t4pkur7p) |
| [G5](https://docs.google.com/document/d/1flDt2vKlwQ_82cwowFYN6X6sJmXbqcUe/edit#heading=h.9fqst5chai2b) (+) (B) | SaPI 10-2 A  (standard) | [*Klebsiella pneumoniae*](https://docs.google.com/document/d/1flDt2vKlwQ_82cwowFYN6X6sJmXbqcUe/edit#heading=h.q6qq3vhxp2hf) | [1.91](https://docs.google.com/document/d/1flDt2vKlwQ_82cwowFYN6X6sJmXbqcUe/edit#heading=h.7362t4pkur7p) | [*Klebsiella pneumoniae*](https://docs.google.com/document/d/1flDt2vKlwQ_82cwowFYN6X6sJmXbqcUe/edit#heading=h.3dag4ul9oqrm) | [1.86](https://docs.google.com/document/d/1flDt2vKlwQ_82cwowFYN6X6sJmXbqcUe/edit#heading=h.7362t4pkur7p) |
| [G6](https://docs.google.com/document/d/1flDt2vKlwQ_82cwowFYN6X6sJmXbqcUe/edit#heading=h.ear5h76gtkx4) (+) (B) | 8HFI 10-1 B  (standard) | [*Klebsiella pneumoniae*](https://docs.google.com/document/d/1flDt2vKlwQ_82cwowFYN6X6sJmXbqcUe/edit#heading=h.q6qq3vhxp2hf) | [1.76](https://docs.google.com/document/d/1flDt2vKlwQ_82cwowFYN6X6sJmXbqcUe/edit#heading=h.7362t4pkur7p) | No Organism Identification Possible | [1.57](https://docs.google.com/document/d/1flDt2vKlwQ_82cwowFYN6X6sJmXbqcUe/edit#heading=h.7362t4pkur7p) |
| [G7](https://docs.google.com/document/d/1flDt2vKlwQ_82cwowFYN6X6sJmXbqcUe/edit#heading=h.acqteihcoekt) (+++) (A) | SaPTI 10-1 A  (standard) | [*Enterobacter bugandensis*](https://docs.google.com/document/d/1flDt2vKlwQ_82cwowFYN6X6sJmXbqcUe/edit#heading=h.au2fqjxehc0v) | [2.24](https://docs.google.com/document/d/1flDt2vKlwQ_82cwowFYN6X6sJmXbqcUe/edit#heading=h.7362t4pkur7p) | [*Enterobacter bugandensis*](https://docs.google.com/document/d/1flDt2vKlwQ_82cwowFYN6X6sJmXbqcUe/edit#heading=h.2g1smts8z1ks) | [2.23](https://docs.google.com/document/d/1flDt2vKlwQ_82cwowFYN6X6sJmXbqcUe/edit#heading=h.7362t4pkur7p) |
| [G8](https://docs.google.com/document/d/1flDt2vKlwQ_82cwowFYN6X6sJmXbqcUe/edit#heading=h.brv0xf6j9l3q)  (-) (B) | SaPTI 10-1 B  (standard) | No Organism Identification Possible | [1.61](https://docs.google.com/document/d/1flDt2vKlwQ_82cwowFYN6X6sJmXbqcUe/edit#heading=h.7362t4pkur7p) | No Organism Identification Possible | [1.47](https://docs.google.com/document/d/1flDt2vKlwQ_82cwowFYN6X6sJmXbqcUe/edit#heading=h.7362t4pkur7p) |
| [G9](https://docs.google.com/document/d/1flDt2vKlwQ_82cwowFYN6X6sJmXbqcUe/edit#heading=h.mmu864b8zszt) (+++) (A) | MMF 10-4  (standard) | [*Klebsiella pneumoniae*](https://docs.google.com/document/d/1flDt2vKlwQ_82cwowFYN6X6sJmXbqcUe/edit#heading=h.q6qq3vhxp2hf) | [2.06](https://docs.google.com/document/d/1flDt2vKlwQ_82cwowFYN6X6sJmXbqcUe/edit#heading=h.7362t4pkur7p) | [*Klebsiella pneumoniae*](https://docs.google.com/document/d/1flDt2vKlwQ_82cwowFYN6X6sJmXbqcUe/edit#heading=h.tqbu6sne82mz) | [1.99](https://docs.google.com/document/d/1flDt2vKlwQ_82cwowFYN6X6sJmXbqcUe/edit#heading=h.7362t4pkur7p) |
| [G10](https://docs.google.com/document/d/1flDt2vKlwQ_82cwowFYN6X6sJmXbqcUe/edit#heading=h.k6mt035wwg7v) (+) (B) | SaPTI 10-1 B  (standard) | [*Mixta calida*](https://docs.google.com/document/d/1flDt2vKlwQ_82cwowFYN6X6sJmXbqcUe/edit#heading=h.hg87uwajnjvu) | [1.79](https://docs.google.com/document/d/1flDt2vKlwQ_82cwowFYN6X6sJmXbqcUe/edit#heading=h.7362t4pkur7p) | [*Mixta calida*](https://docs.google.com/document/d/1flDt2vKlwQ_82cwowFYN6X6sJmXbqcUe/edit#heading=h.e8u0fed4n8bz) | [1.74](https://docs.google.com/document/d/1flDt2vKlwQ_82cwowFYN6X6sJmXbqcUe/edit#heading=h.7362t4pkur7p) |
| [G11](https://docs.google.com/document/d/1flDt2vKlwQ_82cwowFYN6X6sJmXbqcUe/edit#heading=h.wa4d7j3idu5n) (+) (B) | NaCO 10-1  (standard) | [*Enterobacter hormaechei*](https://docs.google.com/document/d/1flDt2vKlwQ_82cwowFYN6X6sJmXbqcUe/edit#heading=h.3usowun49u5w) | [1.99](https://docs.google.com/document/d/1flDt2vKlwQ_82cwowFYN6X6sJmXbqcUe/edit#heading=h.7362t4pkur7p) | [*Enterobacter hormaechei*](https://docs.google.com/document/d/1flDt2vKlwQ_82cwowFYN6X6sJmXbqcUe/edit#heading=h.m6x48h2lfc1e) | [1.97](https://docs.google.com/document/d/1flDt2vKlwQ_82cwowFYN6X6sJmXbqcUe/edit#heading=h.7362t4pkur7p) |
| [G12](https://docs.google.com/document/d/1flDt2vKlwQ_82cwowFYN6X6sJmXbqcUe/edit#heading=h.yrtq3qos8s4a) (+++) (A) | BRFG 10-4 B  (standard) | [*Klebsiella pneumoniae*](https://docs.google.com/document/d/1flDt2vKlwQ_82cwowFYN6X6sJmXbqcUe/edit#heading=h.q6qq3vhxp2hf) | [2.30](https://docs.google.com/document/d/1flDt2vKlwQ_82cwowFYN6X6sJmXbqcUe/edit#heading=h.7362t4pkur7p) | [*Klebsiella pneumoniae*](https://docs.google.com/document/d/1flDt2vKlwQ_82cwowFYN6X6sJmXbqcUe/edit#heading=h.srzgkoeuu3tz) | [2.28](https://docs.google.com/document/d/1flDt2vKlwQ_82cwowFYN6X6sJmXbqcUe/edit#heading=h.7362t4pkur7p) |
| [H1](https://docs.google.com/document/d/1flDt2vKlwQ_82cwowFYN6X6sJmXbqcUe/edit#heading=h.grdve7jlm7gs) (+++) (A) | DRFG 10-4 A  (standard) | [*Klebsiella pneumoniae*](https://docs.google.com/document/d/1flDt2vKlwQ_82cwowFYN6X6sJmXbqcUe/edit#heading=h.srzgkoeuu3tz) | [2.14](https://docs.google.com/document/d/1flDt2vKlwQ_82cwowFYN6X6sJmXbqcUe/edit#heading=h.7362t4pkur7p) | [*Klebsiella pneumoniae*](https://docs.google.com/document/d/1flDt2vKlwQ_82cwowFYN6X6sJmXbqcUe/edit#heading=h.q6qq3vhxp2hf) | [2.13](https://docs.google.com/document/d/1flDt2vKlwQ_82cwowFYN6X6sJmXbqcUe/edit#heading=h.7362t4pkur7p) |
| [H2](https://docs.google.com/document/d/1flDt2vKlwQ_82cwowFYN6X6sJmXbqcUe/edit#heading=h.u00hea6f27q2) (+) (B) | MMFO 10-3 B  (standard) | [*Klebsiella pneumoniae*](https://docs.google.com/document/d/1flDt2vKlwQ_82cwowFYN6X6sJmXbqcUe/edit#heading=h.q6qq3vhxp2hf) | [1.91](https://docs.google.com/document/d/1flDt2vKlwQ_82cwowFYN6X6sJmXbqcUe/edit#heading=h.7362t4pkur7p) | No Organism Identification Possible | [1.69](https://docs.google.com/document/d/1flDt2vKlwQ_82cwowFYN6X6sJmXbqcUe/edit#heading=h.7362t4pkur7p) |
| [H3](https://docs.google.com/document/d/1flDt2vKlwQ_82cwowFYN6X6sJmXbqcUe/edit#heading=h.lkungnbbg3ze) (+) (B) | MMFO 10-3 A  (standard) | [*Klebsiella pneumoniae*](https://docs.google.com/document/d/1flDt2vKlwQ_82cwowFYN6X6sJmXbqcUe/edit#heading=h.q6qq3vhxp2hf) | [1.88](https://docs.google.com/document/d/1flDt2vKlwQ_82cwowFYN6X6sJmXbqcUe/edit#heading=h.7362t4pkur7p) | [*Klebsiella pneumoniae*](https://docs.google.com/document/d/1flDt2vKlwQ_82cwowFYN6X6sJmXbqcUe/edit#heading=h.3dag4ul9oqrm) | [1.74](https://docs.google.com/document/d/1flDt2vKlwQ_82cwowFYN6X6sJmXbqcUe/edit#heading=h.7362t4pkur7p) |
| [H4](https://docs.google.com/document/d/1flDt2vKlwQ_82cwowFYN6X6sJmXbqcUe/edit#heading=h.5mo4nw3oshjw) (+++) (A) | MPIT1 10-3  (standard) | [*Klebsiella pneumoniae*](https://docs.google.com/document/d/1flDt2vKlwQ_82cwowFYN6X6sJmXbqcUe/edit#heading=h.q6qq3vhxp2hf) | [2.15](https://docs.google.com/document/d/1flDt2vKlwQ_82cwowFYN6X6sJmXbqcUe/edit#heading=h.7362t4pkur7p) | [*Klebsiella pneumoniae*](https://docs.google.com/document/d/1flDt2vKlwQ_82cwowFYN6X6sJmXbqcUe/edit#heading=h.odorjocrwyka) | [2.01](https://docs.google.com/document/d/1flDt2vKlwQ_82cwowFYN6X6sJmXbqcUe/edit#heading=h.7362t4pkur7p) |
| [H5](https://docs.google.com/document/d/1flDt2vKlwQ_82cwowFYN6X6sJmXbqcUe/edit#heading=h.pg57k0xfyviy) (+++) (A) | MMFG 10-2 A  (standard) | [*Acinetobacter baumannii*](https://docs.google.com/document/d/1flDt2vKlwQ_82cwowFYN6X6sJmXbqcUe/edit#heading=h.5jnmspakdo75) | [2.07](https://docs.google.com/document/d/1flDt2vKlwQ_82cwowFYN6X6sJmXbqcUe/edit#heading=h.7362t4pkur7p) | [*Acinetobacter baumannii*](https://docs.google.com/document/d/1flDt2vKlwQ_82cwowFYN6X6sJmXbqcUe/edit#heading=h.xp5azwfixjx) | [2.06](https://docs.google.com/document/d/1flDt2vKlwQ_82cwowFYN6X6sJmXbqcUe/edit#heading=h.7362t4pkur7p) |
| [H6](https://docs.google.com/document/d/1flDt2vKlwQ_82cwowFYN6X6sJmXbqcUe/edit#heading=h.z433vp337awa) (+) (B) | TFO B  (standard) | [*Enterobacter cloacae*](https://docs.google.com/document/d/1flDt2vKlwQ_82cwowFYN6X6sJmXbqcUe/edit#heading=h.og79vjor5prn) | [1.84](https://docs.google.com/document/d/1flDt2vKlwQ_82cwowFYN6X6sJmXbqcUe/edit#heading=h.7362t4pkur7p) | [*Enterobacter cloacae*](https://docs.google.com/document/d/1flDt2vKlwQ_82cwowFYN6X6sJmXbqcUe/edit#heading=h.up67ak3r6y1) | [1.82](https://docs.google.com/document/d/1flDt2vKlwQ_82cwowFYN6X6sJmXbqcUe/edit#heading=h.7362t4pkur7p) |
| [H7](https://docs.google.com/document/d/1flDt2vKlwQ_82cwowFYN6X6sJmXbqcUe/edit#heading=h.25skkha2mkdb)  (+) (B) | MMCG A  (standard) | *Klebsiella aerogenes* | [1.](https://docs.google.com/document/d/1flDt2vKlwQ_82cwowFYN6X6sJmXbqcUe/edit#heading=h.7362t4pkur7p)92 | *Klebsiella aerogenes* | [1.](https://docs.google.com/document/d/1flDt2vKlwQ_82cwowFYN6X6sJmXbqcUe/edit#heading=h.7362t4pkur7p)88 |
| [H8](https://docs.google.com/document/d/1flDt2vKlwQ_82cwowFYN6X6sJmXbqcUe/edit#heading=h.hjqr7fr7693b)  (+) (B) | NAFO 10-1 C  (standard) | *Klebsiella variicola* | [1.](https://docs.google.com/document/d/1flDt2vKlwQ_82cwowFYN6X6sJmXbqcUe/edit#heading=h.7362t4pkur7p)79 | *Klebsiella variicola* | [1.](https://docs.google.com/document/d/1flDt2vKlwQ_82cwowFYN6X6sJmXbqcUe/edit#heading=h.7362t4pkur7p)72 |
| [H9](https://docs.google.com/document/d/1flDt2vKlwQ_82cwowFYN6X6sJmXbqcUe/edit#heading=h.3sh4e9dkhvva)  (-) (C) | SaFI A  (standard) | no peaks found | [0.00](https://docs.google.com/document/d/1flDt2vKlwQ_82cwowFYN6X6sJmXbqcUe/edit#heading=h.7362t4pkur7p) | no peaks found | [0.00](https://docs.google.com/document/d/1flDt2vKlwQ_82cwowFYN6X6sJmXbqcUe/edit#heading=h.7362t4pkur7p) |
| [H10](https://docs.google.com/document/d/1flDt2vKlwQ_82cwowFYN6X6sJmXbqcUe/edit#heading=h.kdt4p7cmqz6h) (+++) (A) | SaFI B  (standard) | [*Klebsiella pneumoniae*](https://docs.google.com/document/d/1flDt2vKlwQ_82cwowFYN6X6sJmXbqcUe/edit#heading=h.q6qq3vhxp2hf) | [2.11](https://docs.google.com/document/d/1flDt2vKlwQ_82cwowFYN6X6sJmXbqcUe/edit#heading=h.7362t4pkur7p) | [*Klebsiella pneumoniae*](https://docs.google.com/document/d/1flDt2vKlwQ_82cwowFYN6X6sJmXbqcUe/edit#heading=h.l8o9l3joithh) | [2.06](https://docs.google.com/document/d/1flDt2vKlwQ_82cwowFYN6X6sJmXbqcUe/edit#heading=h.7362t4pkur7p) |
| [H11](https://docs.google.com/document/d/1flDt2vKlwQ_82cwowFYN6X6sJmXbqcUe/edit#heading=h.x9sgd9657wm5) (+++) (B) | SaFI D  (standard) | [*Enterobacter asburiae*](https://docs.google.com/document/d/1flDt2vKlwQ_82cwowFYN6X6sJmXbqcUe/edit#heading=h.bpu73ci3n5u2) | [2.18](https://docs.google.com/document/d/1flDt2vKlwQ_82cwowFYN6X6sJmXbqcUe/edit#heading=h.7362t4pkur7p) | [*Enterobacter kobei*](https://docs.google.com/document/d/1flDt2vKlwQ_82cwowFYN6X6sJmXbqcUe/edit#heading=h.6pil7oydtogv) | [2.11](https://docs.google.com/document/d/1flDt2vKlwQ_82cwowFYN6X6sJmXbqcUe/edit#heading=h.7362t4pkur7p) |
| [H12](https://docs.google.com/document/d/1flDt2vKlwQ_82cwowFYN6X6sJmXbqcUe/edit#heading=h.sjvg46jd17lu) (+) (B) | MMCO 10-1 A  (standard) | [*Klebsiella pneumoniae*](https://docs.google.com/document/d/1flDt2vKlwQ_82cwowFYN6X6sJmXbqcUe/edit#heading=h.q6qq3vhxp2hf) | [1.94](https://docs.google.com/document/d/1flDt2vKlwQ_82cwowFYN6X6sJmXbqcUe/edit#heading=h.7362t4pkur7p) | [*Klebsiella pneumoniae*](https://docs.google.com/document/d/1flDt2vKlwQ_82cwowFYN6X6sJmXbqcUe/edit#heading=h.l8o9l3joithh) | [1.73](https://docs.google.com/document/d/1flDt2vKlwQ_82cwowFYN6X6sJmXbqcUe/edit#heading=h.7362t4pkur7p) |
| [A1](https://docs.google.com/document/d/13sR9l8TvqBdnMS5XbT1vdYjt-tTESQPp/edit#heading=h.8lsxze5tu970) (+++) (A) | SAFG2 10-1  (standard) | [*Klebsiella pneumoniae*](https://docs.google.com/document/d/13sR9l8TvqBdnMS5XbT1vdYjt-tTESQPp/edit#heading=h.7f69eol1axqu) | [2.03](https://docs.google.com/document/d/13sR9l8TvqBdnMS5XbT1vdYjt-tTESQPp/edit#heading=h.fx984lxbj9qx) | [*Klebsiella pneumoniae*](https://docs.google.com/document/d/13sR9l8TvqBdnMS5XbT1vdYjt-tTESQPp/edit#heading=h.snknyjj68yuc) | [1.97](https://docs.google.com/document/d/13sR9l8TvqBdnMS5XbT1vdYjt-tTESQPp/edit#heading=h.fx984lxbj9qx) |
| [A2](https://docs.google.com/document/d/13sR9l8TvqBdnMS5XbT1vdYjt-tTESQPp/edit#heading=h.w33kj441bh01)  (-) (C) | A2NACI 10-1 A  (standard) | no peaks found | [0.00](https://docs.google.com/document/d/13sR9l8TvqBdnMS5XbT1vdYjt-tTESQPp/edit#heading=h.fx984lxbj9qx) | no peaks found | [0.00](https://docs.google.com/document/d/13sR9l8TvqBdnMS5XbT1vdYjt-tTESQPp/edit#heading=h.fx984lxbj9qx) |
| [A3](https://docs.google.com/document/d/13sR9l8TvqBdnMS5XbT1vdYjt-tTESQPp/edit#heading=h.cni2kgqio8jh) (+++) (A) | NACI 10-1 B  (standard) | [*Klebsiella pneumoniae*](https://docs.google.com/document/d/13sR9l8TvqBdnMS5XbT1vdYjt-tTESQPp/edit#heading=h.snknyjj68yuc) | [2.20](https://docs.google.com/document/d/13sR9l8TvqBdnMS5XbT1vdYjt-tTESQPp/edit#heading=h.fx984lxbj9qx) | [*Klebsiella pneumoniae*](https://docs.google.com/document/d/13sR9l8TvqBdnMS5XbT1vdYjt-tTESQPp/edit#heading=h.dso9ibhkjs6s) | [2.15](https://docs.google.com/document/d/13sR9l8TvqBdnMS5XbT1vdYjt-tTESQPp/edit#heading=h.fx984lxbj9qx) |
| [A4](https://docs.google.com/document/d/13sR9l8TvqBdnMS5XbT1vdYjt-tTESQPp/edit#heading=h.iu4hk1ysttb) (+++) (A) | A4ZCO 10-  1 A  (standard) | [*Enterobacter bugandensis*](https://docs.google.com/document/d/13sR9l8TvqBdnMS5XbT1vdYjt-tTESQPp/edit#heading=h.dkrd718qsnn) | [2.11](https://docs.google.com/document/d/13sR9l8TvqBdnMS5XbT1vdYjt-tTESQPp/edit#heading=h.fx984lxbj9qx) | [*Enterobacter bugandensis*](https://docs.google.com/document/d/13sR9l8TvqBdnMS5XbT1vdYjt-tTESQPp/edit#heading=h.siobdf8l3okv) | [2.09](https://docs.google.com/document/d/13sR9l8TvqBdnMS5XbT1vdYjt-tTESQPp/edit#heading=h.fx984lxbj9qx) |
| [A5](https://docs.google.com/document/d/13sR9l8TvqBdnMS5XbT1vdYjt-tTESQPp/edit#heading=h.l82p6sl39l4v) (+) (B) | MCFI2 10-  3 A  (standard) | [*Acinetobacter nosocomialis*](https://docs.google.com/document/d/13sR9l8TvqBdnMS5XbT1vdYjt-tTESQPp/edit#heading=h.4znm6cahu39u) | [1.70](https://docs.google.com/document/d/13sR9l8TvqBdnMS5XbT1vdYjt-tTESQPp/edit#heading=h.fx984lxbj9qx) | No Organism Identification Possible | [1.64](https://docs.google.com/document/d/13sR9l8TvqBdnMS5XbT1vdYjt-tTESQPp/edit#heading=h.fx984lxbj9qx) |
| [A6](https://docs.google.com/document/d/13sR9l8TvqBdnMS5XbT1vdYjt-tTESQPp/edit#heading=h.p1zmacuoaoi3)  (-) (C) | MCFI2 10-  3 B  (standard) | no peaks found | [0.00](https://docs.google.com/document/d/13sR9l8TvqBdnMS5XbT1vdYjt-tTESQPp/edit#heading=h.fx984lxbj9qx) | no peaks found | [0.00](https://docs.google.com/document/d/13sR9l8TvqBdnMS5XbT1vdYjt-tTESQPp/edit#heading=h.fx984lxbj9qx) |
| [A7](https://docs.google.com/document/d/13sR9l8TvqBdnMS5XbT1vdYjt-tTESQPp/edit#heading=h.yyuc0a4utroy) (+) (B) | MCFO 10-  3 A  (standard) | [*Enterobacter kobei*](https://docs.google.com/document/d/13sR9l8TvqBdnMS5XbT1vdYjt-tTESQPp/edit#heading=h.uqvuv1c7v39l) | [1.88](https://docs.google.com/document/d/13sR9l8TvqBdnMS5XbT1vdYjt-tTESQPp/edit#heading=h.fx984lxbj9qx) | No Organism Identification Possible | [1.64](https://docs.google.com/document/d/13sR9l8TvqBdnMS5XbT1vdYjt-tTESQPp/edit#heading=h.fx984lxbj9qx) |
| [A8](https://docs.google.com/document/d/13sR9l8TvqBdnMS5XbT1vdYjt-tTESQPp/edit#heading=h.iy0lo8vt2ydw)  (-) (C) | ACI 10-  4 A  (standard) | no peaks found | [0.00](https://docs.google.com/document/d/13sR9l8TvqBdnMS5XbT1vdYjt-tTESQPp/edit#heading=h.fx984lxbj9qx) | no peaks found | [0.00](https://docs.google.com/document/d/13sR9l8TvqBdnMS5XbT1vdYjt-tTESQPp/edit#heading=h.fx984lxbj9qx) |
| [A9](https://docs.google.com/document/d/13sR9l8TvqBdnMS5XbT1vdYjt-tTESQPp/edit#heading=h.u8enb3mgmucu)  (+) (B) | ACI 10-  4 B  (standard) | *Klebsiella pneumoniae* | [1.](https://docs.google.com/document/d/13sR9l8TvqBdnMS5XbT1vdYjt-tTESQPp/edit#heading=h.fx984lxbj9qx)93 | *Klebsiella pneumoniae* | [1.](https://docs.google.com/document/d/13sR9l8TvqBdnMS5XbT1vdYjt-tTESQPp/edit#heading=h.fx984lxbj9qx)90 |
| [A10](https://docs.google.com/document/d/13sR9l8TvqBdnMS5XbT1vdYjt-tTESQPp/edit#heading=h.m9vpy18k5ram) (+) (B) | MCFI 10-  3 A  (standard) | [*Klebsiella pneumoniae*](https://docs.google.com/document/d/13sR9l8TvqBdnMS5XbT1vdYjt-tTESQPp/edit#heading=h.snknyjj68yuc) | [1.94](https://docs.google.com/document/d/13sR9l8TvqBdnMS5XbT1vdYjt-tTESQPp/edit#heading=h.fx984lxbj9qx) | [*Klebsiella pneumoniae*](https://docs.google.com/document/d/13sR9l8TvqBdnMS5XbT1vdYjt-tTESQPp/edit#heading=h.7f69eol1axqu) | [1.73](https://docs.google.com/document/d/13sR9l8TvqBdnMS5XbT1vdYjt-tTESQPp/edit#heading=h.fx984lxbj9qx) |
| [A11](https://docs.google.com/document/d/13sR9l8TvqBdnMS5XbT1vdYjt-tTESQPp/edit#heading=h.qmaxyhujq9m) (-) (C) | MCFI 10-  3 B  (standard) | no peaks found | [0.00](https://docs.google.com/document/d/13sR9l8TvqBdnMS5XbT1vdYjt-tTESQPp/edit#heading=h.fx984lxbj9qx) | no peaks found | [0.00](https://docs.google.com/document/d/13sR9l8TvqBdnMS5XbT1vdYjt-tTESQPp/edit#heading=h.fx984lxbj9qx) |
| [A12](https://docs.google.com/document/d/13sR9l8TvqBdnMS5XbT1vdYjt-tTESQPp/edit#heading=h.hi9hxg83a2ae) (+) (B) | NAFGA 10-1  (standard) | [*Klebsiella pneumoniae*](https://docs.google.com/document/d/13sR9l8TvqBdnMS5XbT1vdYjt-tTESQPp/edit#heading=h.7f69eol1axqu) | [1.95](https://docs.google.com/document/d/13sR9l8TvqBdnMS5XbT1vdYjt-tTESQPp/edit#heading=h.fx984lxbj9qx) | [*Klebsiella variicola*](https://docs.google.com/document/d/13sR9l8TvqBdnMS5XbT1vdYjt-tTESQPp/edit#heading=h.3oq5g43w6bwp) | [1.90](https://docs.google.com/document/d/13sR9l8TvqBdnMS5XbT1vdYjt-tTESQPp/edit#heading=h.fx984lxbj9qx) |
| [B1](https://docs.google.com/document/d/13sR9l8TvqBdnMS5XbT1vdYjt-tTESQPp/edit#heading=h.cn9fzi2ococ6) (+++) (A) | NAFG 10-  4 B  (standard) | [*Klebsiella pneumoniae*](https://docs.google.com/document/d/13sR9l8TvqBdnMS5XbT1vdYjt-tTESQPp/edit#heading=h.snknyjj68yuc) | [2.09](https://docs.google.com/document/d/13sR9l8TvqBdnMS5XbT1vdYjt-tTESQPp/edit#heading=h.fx984lxbj9qx) | [*Klebsiella pneumoniae*](https://docs.google.com/document/d/13sR9l8TvqBdnMS5XbT1vdYjt-tTESQPp/edit#heading=h.qc77odrrq8h3) | [1.98](https://docs.google.com/document/d/13sR9l8TvqBdnMS5XbT1vdYjt-tTESQPp/edit#heading=h.fx984lxbj9qx) |
| [B2](https://docs.google.com/document/d/13sR9l8TvqBdnMS5XbT1vdYjt-tTESQPp/edit#heading=h.vpgawn6rv5ge) (+++) (A) | SACG 10-1  (standard) | [*Enterobacter cloacae*](https://docs.google.com/document/d/13sR9l8TvqBdnMS5XbT1vdYjt-tTESQPp/edit#heading=h.isq7utg05rc2) | [2.18](https://docs.google.com/document/d/13sR9l8TvqBdnMS5XbT1vdYjt-tTESQPp/edit#heading=h.fx984lxbj9qx) | [*Enterobacter cloacae*](https://docs.google.com/document/d/13sR9l8TvqBdnMS5XbT1vdYjt-tTESQPp/edit#heading=h.rlopleg70bxt) | [2.10](https://docs.google.com/document/d/13sR9l8TvqBdnMS5XbT1vdYjt-tTESQPp/edit#heading=h.fx984lxbj9qx) |
| [B3](https://docs.google.com/document/d/13sR9l8TvqBdnMS5XbT1vdYjt-tTESQPp/edit#heading=h.fk3o65eyzyvf) (+) (B) | SRAFO 10-4 B  (standard) | [*Escherichia coli*](https://docs.google.com/document/d/13sR9l8TvqBdnMS5XbT1vdYjt-tTESQPp/edit#heading=h.c2dtl1cwnf9u) | [1.84](https://docs.google.com/document/d/13sR9l8TvqBdnMS5XbT1vdYjt-tTESQPp/edit#heading=h.fx984lxbj9qx) | No Organism Identification Possible | [1.63](https://docs.google.com/document/d/13sR9l8TvqBdnMS5XbT1vdYjt-tTESQPp/edit#heading=h.fx984lxbj9qx) |
| [B4](https://docs.google.com/document/d/13sR9l8TvqBdnMS5XbT1vdYjt-tTESQPp/edit#heading=h.957j8g811x7o)  (-) (C) | SRACG 10-3 A  (standard) | no peaks found | [0.00](https://docs.google.com/document/d/13sR9l8TvqBdnMS5XbT1vdYjt-tTESQPp/edit#heading=h.fx984lxbj9qx) | no peaks found | [0.00](https://docs.google.com/document/d/13sR9l8TvqBdnMS5XbT1vdYjt-tTESQPp/edit#heading=h.fx984lxbj9qx) |
| [B5](https://docs.google.com/document/d/13sR9l8TvqBdnMS5XbT1vdYjt-tTESQPp/edit#heading=h.c09scfp806ed) (+) (B) | SRACG 10-3  (standard) | [*Enterobacter cloacae*](https://docs.google.com/document/d/13sR9l8TvqBdnMS5XbT1vdYjt-tTESQPp/edit#heading=h.4gb36rddu8lk) | [1.91](https://docs.google.com/document/d/13sR9l8TvqBdnMS5XbT1vdYjt-tTESQPp/edit#heading=h.fx984lxbj9qx) | [*Enterobacter cloacae*](https://docs.google.com/document/d/13sR9l8TvqBdnMS5XbT1vdYjt-tTESQPp/edit#heading=h.rlopleg70bxt) | [1.90](https://docs.google.com/document/d/13sR9l8TvqBdnMS5XbT1vdYjt-tTESQPp/edit#heading=h.fx984lxbj9qx) |
| [B8](https://docs.google.com/document/d/13sR9l8TvqBdnMS5XbT1vdYjt-tTESQPp/edit#heading=h.snaf6kitjlag) (+) (B) | RAFG 10-1 B  (standard) | [*Klebsiella pneumoniae*](https://docs.google.com/document/d/13sR9l8TvqBdnMS5XbT1vdYjt-tTESQPp/edit#heading=h.snknyjj68yuc) | [1.86](https://docs.google.com/document/d/13sR9l8TvqBdnMS5XbT1vdYjt-tTESQPp/edit#heading=h.fx984lxbj9qx) | [*Klebsiella pneumoniae*](https://docs.google.com/document/d/13sR9l8TvqBdnMS5XbT1vdYjt-tTESQPp/edit#heading=h.qc77odrrq8h3) | [1.77](https://docs.google.com/document/d/13sR9l8TvqBdnMS5XbT1vdYjt-tTESQPp/edit#heading=h.fx984lxbj9qx) |
| [B9](https://docs.google.com/document/d/13sR9l8TvqBdnMS5XbT1vdYjt-tTESQPp/edit#heading=h.u61wxlj63tex)  (+++) (A) | TFI 10-2 B  (standard) | *Klebsiella pneumoniae* | 2.07 | *Klebsiella pneumoniae* | 1.89 |
| [B10](https://docs.google.com/document/d/13sR9l8TvqBdnMS5XbT1vdYjt-tTESQPp/edit#heading=h.8c1x5ce4fhaf) (+++) (A) | SAPT G 10-3  (standard) | *Klebsiella pneumoniae* | 2.44 | *Klebsiella pneumoniae* | 2.03 |
| [B11](https://docs.google.com/document/d/13sR9l8TvqBdnMS5XbT1vdYjt-tTESQPp/edit#heading=h.f9w5taeji0b1) (+) (B) | MMCO 10-1 C  (standard) | *Klebsiella pneumoniae* | [1.](https://docs.google.com/document/d/13sR9l8TvqBdnMS5XbT1vdYjt-tTESQPp/edit#heading=h.fx984lxbj9qx)82 | *Klebsiella pneumoniae* | [1.](https://docs.google.com/document/d/13sR9l8TvqBdnMS5XbT1vdYjt-tTESQPp/edit#heading=h.fx984lxbj9qx)75 |
| [B12](https://docs.google.com/document/d/13sR9l8TvqBdnMS5XbT1vdYjt-tTESQPp/edit#heading=h.s8kucdhxapek) (+) (B) | SHFG 10-1  (standard) | [*Enterobacter kobei*](https://docs.google.com/document/d/13sR9l8TvqBdnMS5XbT1vdYjt-tTESQPp/edit#heading=h.uqvuv1c7v39l) | [1.90](https://docs.google.com/document/d/13sR9l8TvqBdnMS5XbT1vdYjt-tTESQPp/edit#heading=h.fx984lxbj9qx) | [*Enterobacter asburiae*](https://docs.google.com/document/d/13sR9l8TvqBdnMS5XbT1vdYjt-tTESQPp/edit#heading=h.mkqiybcq5rnn) | [1.71](https://docs.google.com/document/d/13sR9l8TvqBdnMS5XbT1vdYjt-tTESQPp/edit#heading=h.fx984lxbj9qx) |
| [C1](https://docs.google.com/document/d/13sR9l8TvqBdnMS5XbT1vdYjt-tTESQPp/edit#heading=h.p7qbn14b2z5r)  (+) (B) | NACG 10-1  (standard) | *Acinetobacter baumannii* | [1.](https://docs.google.com/document/d/13sR9l8TvqBdnMS5XbT1vdYjt-tTESQPp/edit#heading=h.fx984lxbj9qx)80 | *Acinetobacter baumannii* | [1.](https://docs.google.com/document/d/13sR9l8TvqBdnMS5XbT1vdYjt-tTESQPp/edit#heading=h.fx984lxbj9qx)72 |
| [C2](https://docs.google.com/document/d/13sR9l8TvqBdnMS5XbT1vdYjt-tTESQPp/edit#heading=h.j4itivxs3e9d) (+) (B) | SAFI 10-4  (standard) | [*Enterobacter hormaechei*](https://docs.google.com/document/d/13sR9l8TvqBdnMS5XbT1vdYjt-tTESQPp/edit#heading=h.p7dbd1wdaz79) | [1.88](https://docs.google.com/document/d/13sR9l8TvqBdnMS5XbT1vdYjt-tTESQPp/edit#heading=h.fx984lxbj9qx) | [*Enterobacter hormaechei*](https://docs.google.com/document/d/13sR9l8TvqBdnMS5XbT1vdYjt-tTESQPp/edit#heading=h.38j3hp2okge2) | [1.71](https://docs.google.com/document/d/13sR9l8TvqBdnMS5XbT1vdYjt-tTESQPp/edit#heading=h.fx984lxbj9qx) |
| [C3](https://docs.google.com/document/d/13sR9l8TvqBdnMS5XbT1vdYjt-tTESQPp/edit#heading=h.op0q3iomldsm) (+++) (A) | SRAFG 10-4 A  (standard) | [*Klebsiella pneumoniae*](https://docs.google.com/document/d/13sR9l8TvqBdnMS5XbT1vdYjt-tTESQPp/edit#heading=h.snknyjj68yuc) | [2.00](https://docs.google.com/document/d/13sR9l8TvqBdnMS5XbT1vdYjt-tTESQPp/edit#heading=h.fx984lxbj9qx) | [*Klebsiella pneumoniae*](https://docs.google.com/document/d/13sR9l8TvqBdnMS5XbT1vdYjt-tTESQPp/edit#heading=h.qc77odrrq8h3) | [1.84](https://docs.google.com/document/d/13sR9l8TvqBdnMS5XbT1vdYjt-tTESQPp/edit#heading=h.fx984lxbj9qx) |
| [C4](https://docs.google.com/document/d/13sR9l8TvqBdnMS5XbT1vdYjt-tTESQPp/edit#heading=h.bmp0nh5alhzb) (+++) (A) | SRAG 10-2 A  (standard) | [*Klebsiella pneumoniae*](https://docs.google.com/document/d/13sR9l8TvqBdnMS5XbT1vdYjt-tTESQPp/edit#heading=h.snknyjj68yuc) | [2.18](https://docs.google.com/document/d/13sR9l8TvqBdnMS5XbT1vdYjt-tTESQPp/edit#heading=h.fx984lxbj9qx) | [*Klebsiella pneumoniae*](https://docs.google.com/document/d/13sR9l8TvqBdnMS5XbT1vdYjt-tTESQPp/edit#heading=h.7f69eol1axqu) | [2.03](https://docs.google.com/document/d/13sR9l8TvqBdnMS5XbT1vdYjt-tTESQPp/edit#heading=h.fx984lxbj9qx) |
| [C5](https://docs.google.com/document/d/13sR9l8TvqBdnMS5XbT1vdYjt-tTESQPp/edit#heading=h.55kxbnu087ic) (+++) (A) | SRAG 10-2 B  (standard) | [*Enterobacter roggenkampii*](https://docs.google.com/document/d/13sR9l8TvqBdnMS5XbT1vdYjt-tTESQPp/edit#heading=h.w67041r4e601) | [2.25](https://docs.google.com/document/d/13sR9l8TvqBdnMS5XbT1vdYjt-tTESQPp/edit#heading=h.fx984lxbj9qx) | [*Enterobacter roggenkampii*](https://docs.google.com/document/d/13sR9l8TvqBdnMS5XbT1vdYjt-tTESQPp/edit#heading=h.axqanj3mvr2h) | [2.25](https://docs.google.com/document/d/13sR9l8TvqBdnMS5XbT1vdYjt-tTESQPp/edit#heading=h.fx984lxbj9qx) |
| [C6](https://docs.google.com/document/d/13sR9l8TvqBdnMS5XbT1vdYjt-tTESQPp/edit#heading=h.u8je5dszvs5a)  (+) (B) | OFG3 10-4 B  (standard) | *Enterobacter cloacae* | 1.77 | *Enterobacter cloacae* | 1.51 |
| [C7](https://docs.google.com/document/d/13sR9l8TvqBdnMS5XbT1vdYjt-tTESQPp/edit#heading=h.z2l7ltz6ct4i) (+) (B) | RAFG 10-  1 A  (standard) | [*Klebsiella pneumoniae*](https://docs.google.com/document/d/13sR9l8TvqBdnMS5XbT1vdYjt-tTESQPp/edit#heading=h.snknyjj68yuc) | [1.76](https://docs.google.com/document/d/13sR9l8TvqBdnMS5XbT1vdYjt-tTESQPp/edit#heading=h.fx984lxbj9qx) | No Organism Identification Possible | [1.59](https://docs.google.com/document/d/13sR9l8TvqBdnMS5XbT1vdYjt-tTESQPp/edit#heading=h.fx984lxbj9qx) |
| [C8](https://docs.google.com/document/d/13sR9l8TvqBdnMS5XbT1vdYjt-tTESQPp/edit#heading=h.3960z5gwv70y) (+) (B) | ACO2 10-3  (standard) | [*Enterobacter cloacae*](https://docs.google.com/document/d/13sR9l8TvqBdnMS5XbT1vdYjt-tTESQPp/edit#heading=h.rlopleg70bxt) | [1.83](https://docs.google.com/document/d/13sR9l8TvqBdnMS5XbT1vdYjt-tTESQPp/edit#heading=h.fx984lxbj9qx) | [*Enterobacter cloacae*](https://docs.google.com/document/d/13sR9l8TvqBdnMS5XbT1vdYjt-tTESQPp/edit#heading=h.isq7utg05rc2) | [1.79](https://docs.google.com/document/d/13sR9l8TvqBdnMS5XbT1vdYjt-tTESQPp/edit#heading=h.fx984lxbj9qx) |
| [C9](https://docs.google.com/document/d/13sR9l8TvqBdnMS5XbT1vdYjt-tTESQPp/edit#heading=h.2qiphnaajlz8) (+) (B) | TFO 10-3  (standard) | [*Klebsiella pneumoniae*](https://docs.google.com/document/d/13sR9l8TvqBdnMS5XbT1vdYjt-tTESQPp/edit#heading=h.snknyjj68yuc) | [1.90](https://docs.google.com/document/d/13sR9l8TvqBdnMS5XbT1vdYjt-tTESQPp/edit#heading=h.fx984lxbj9qx) | [*Klebsiella pneumoniae*](https://docs.google.com/document/d/13sR9l8TvqBdnMS5XbT1vdYjt-tTESQPp/edit#heading=h.dso9ibhkjs6s) | [1.75](https://docs.google.com/document/d/13sR9l8TvqBdnMS5XbT1vdYjt-tTESQPp/edit#heading=h.fx984lxbj9qx) |
| [C10](https://docs.google.com/document/d/13sR9l8TvqBdnMS5XbT1vdYjt-tTESQPp/edit#heading=h.3eqmdaqj9yrq) (+) (B) | MEFO 2  10-1 B  (standard) | [*Enterobacter hormaechei*](https://docs.google.com/document/d/13sR9l8TvqBdnMS5XbT1vdYjt-tTESQPp/edit#heading=h.eikpetwlhd3a) | [1.79](https://docs.google.com/document/d/13sR9l8TvqBdnMS5XbT1vdYjt-tTESQPp/edit#heading=h.fx984lxbj9qx) | No Organism Identification Possible | [1.67](https://docs.google.com/document/d/13sR9l8TvqBdnMS5XbT1vdYjt-tTESQPp/edit#heading=h.fx984lxbj9qx) |
| [C11](https://docs.google.com/document/d/13sR9l8TvqBdnMS5XbT1vdYjt-tTESQPp/edit#heading=h.q152803zde11) (+++) (A) | MEFO 2  10-1 C  (standard) | [*Enterobacter cloacae*](https://docs.google.com/document/d/13sR9l8TvqBdnMS5XbT1vdYjt-tTESQPp/edit#heading=h.isq7utg05rc2) | [2.23](https://docs.google.com/document/d/13sR9l8TvqBdnMS5XbT1vdYjt-tTESQPp/edit#heading=h.fx984lxbj9qx) | [*Enterobacter cloacae*](https://docs.google.com/document/d/13sR9l8TvqBdnMS5XbT1vdYjt-tTESQPp/edit#heading=h.rlopleg70bxt) | [2.21](https://docs.google.com/document/d/13sR9l8TvqBdnMS5XbT1vdYjt-tTESQPp/edit#heading=h.fx984lxbj9qx) |
| [C12](https://docs.google.com/document/d/13sR9l8TvqBdnMS5XbT1vdYjt-tTESQPp/edit#heading=h.qi7ia0rtvvq5) (+) (B) | ACG 10-1 B  (standard) | [*Acinetobacter nosocomialis*](https://docs.google.com/document/d/13sR9l8TvqBdnMS5XbT1vdYjt-tTESQPp/edit#heading=h.4znm6cahu39u) | [1.73](https://docs.google.com/document/d/13sR9l8TvqBdnMS5XbT1vdYjt-tTESQPp/edit#heading=h.fx984lxbj9qx) | [*Acinetobacter baumannii*](https://docs.google.com/document/d/13sR9l8TvqBdnMS5XbT1vdYjt-tTESQPp/edit#heading=h.4jw3tw6ifglv) | [1.72](https://docs.google.com/document/d/13sR9l8TvqBdnMS5XbT1vdYjt-tTESQPp/edit#heading=h.fx984lxbj9qx) |
| [D1](https://docs.google.com/document/d/13sR9l8TvqBdnMS5XbT1vdYjt-tTESQPp/edit#heading=h.9s9770wlk6ia) (+++) (A) | ACG 10-1  A  (standard) | [*Escherichia coli*](https://docs.google.com/document/d/13sR9l8TvqBdnMS5XbT1vdYjt-tTESQPp/edit#heading=h.c2dtl1cwnf9u) | [2.14](https://docs.google.com/document/d/13sR9l8TvqBdnMS5XbT1vdYjt-tTESQPp/edit#heading=h.fx984lxbj9qx) | [*Escherichia coli*](https://docs.google.com/document/d/13sR9l8TvqBdnMS5XbT1vdYjt-tTESQPp/edit#heading=h.vy1sv4xln4me) | [2.05](https://docs.google.com/document/d/13sR9l8TvqBdnMS5XbT1vdYjt-tTESQPp/edit#heading=h.fx984lxbj9qx) |
| [D2](https://docs.google.com/document/d/13sR9l8TvqBdnMS5XbT1vdYjt-tTESQPp/edit#heading=h.6l4mdwq5197c) (+++) (A) | SAFO1 10-  4 A  (standard) | [*Klebsiella oxytoca*](https://docs.google.com/document/d/13sR9l8TvqBdnMS5XbT1vdYjt-tTESQPp/edit#heading=h.gn3u3y3zj02h) | [2.13](https://docs.google.com/document/d/13sR9l8TvqBdnMS5XbT1vdYjt-tTESQPp/edit#heading=h.fx984lxbj9qx) | [*Klebsiella oxytoca*](https://docs.google.com/document/d/13sR9l8TvqBdnMS5XbT1vdYjt-tTESQPp/edit#heading=h.4431yageyom2) | [2.09](https://docs.google.com/document/d/13sR9l8TvqBdnMS5XbT1vdYjt-tTESQPp/edit#heading=h.fx984lxbj9qx) |
| [D3](https://docs.google.com/document/d/13sR9l8TvqBdnMS5XbT1vdYjt-tTESQPp/edit#heading=h.7l1k0fquvbvr) (+++) (A) | SAC2G 10-2 A  (standard) | [*Klebsiella aerogenes*](https://docs.google.com/document/d/13sR9l8TvqBdnMS5XbT1vdYjt-tTESQPp/edit#heading=h.f8q3ahrg5sz9) | [2.18](https://docs.google.com/document/d/13sR9l8TvqBdnMS5XbT1vdYjt-tTESQPp/edit#heading=h.fx984lxbj9qx) | [*Klebsiella aerogenes*](https://docs.google.com/document/d/13sR9l8TvqBdnMS5XbT1vdYjt-tTESQPp/edit#heading=h.3gf548pf2su7) | [2.09](https://docs.google.com/document/d/13sR9l8TvqBdnMS5XbT1vdYjt-tTESQPp/edit#heading=h.fx984lxbj9qx) |
| [D4](https://docs.google.com/document/d/13sR9l8TvqBdnMS5XbT1vdYjt-tTESQPp/edit#heading=h.p3k5t5r4o23q) (+++) (A) | SAC2G 10-2 B  (standard) | [*Klebsiella pneumoniae*](https://docs.google.com/document/d/13sR9l8TvqBdnMS5XbT1vdYjt-tTESQPp/edit#heading=h.7f69eol1axqu) | [2.19](https://docs.google.com/document/d/13sR9l8TvqBdnMS5XbT1vdYjt-tTESQPp/edit#heading=h.fx984lxbj9qx) | [*Klebsiella pneumoniae*](https://docs.google.com/document/d/13sR9l8TvqBdnMS5XbT1vdYjt-tTESQPp/edit#heading=h.snknyjj68yuc) | [2.11](https://docs.google.com/document/d/13sR9l8TvqBdnMS5XbT1vdYjt-tTESQPp/edit#heading=h.fx984lxbj9qx) |
| [D5](https://docs.google.com/document/d/13sR9l8TvqBdnMS5XbT1vdYjt-tTESQPp/edit#heading=h.tco3cmbzt0i1) (+++) (A) | SAFO3 10-1 B  (standard) | [*Klebsiella pneumoniae*](https://docs.google.com/document/d/13sR9l8TvqBdnMS5XbT1vdYjt-tTESQPp/edit#heading=h.snknyjj68yuc) | [2.00](https://docs.google.com/document/d/13sR9l8TvqBdnMS5XbT1vdYjt-tTESQPp/edit#heading=h.fx984lxbj9qx) | No Organism Identification Possible | [1.68](https://docs.google.com/document/d/13sR9l8TvqBdnMS5XbT1vdYjt-tTESQPp/edit#heading=h.fx984lxbj9qx) |
| [D6](https://docs.google.com/document/d/13sR9l8TvqBdnMS5XbT1vdYjt-tTESQPp/edit#heading=h.77duwrd6myku) (+) (B) | MMCI  10-3 A  (standard) | [*Klebsiella pneumoniae*](https://docs.google.com/document/d/13sR9l8TvqBdnMS5XbT1vdYjt-tTESQPp/edit#heading=h.dso9ibhkjs6s) | [1.90](https://docs.google.com/document/d/13sR9l8TvqBdnMS5XbT1vdYjt-tTESQPp/edit#heading=h.fx984lxbj9qx) | [*Klebsiella pneumoniae*](https://docs.google.com/document/d/13sR9l8TvqBdnMS5XbT1vdYjt-tTESQPp/edit#heading=h.snknyjj68yuc) | [1.90](https://docs.google.com/document/d/13sR9l8TvqBdnMS5XbT1vdYjt-tTESQPp/edit#heading=h.fx984lxbj9qx) |
| [D7](https://docs.google.com/document/d/13sR9l8TvqBdnMS5XbT1vdYjt-tTESQPp/edit#heading=h.mea7kox5yhhe) (+++) (A) | SAFI3 A  (standard) | [*Klebsiella pneumoniae*](https://docs.google.com/document/d/13sR9l8TvqBdnMS5XbT1vdYjt-tTESQPp/edit#heading=h.snknyjj68yuc) | [2.17](https://docs.google.com/document/d/13sR9l8TvqBdnMS5XbT1vdYjt-tTESQPp/edit#heading=h.fx984lxbj9qx) | [*Klebsiella pneumoniae*](https://docs.google.com/document/d/13sR9l8TvqBdnMS5XbT1vdYjt-tTESQPp/edit#heading=h.7f69eol1axqu) | [2.11](https://docs.google.com/document/d/13sR9l8TvqBdnMS5XbT1vdYjt-tTESQPp/edit#heading=h.fx984lxbj9qx) |
| [D8](https://docs.google.com/document/d/13sR9l8TvqBdnMS5XbT1vdYjt-tTESQPp/edit#heading=h.poasctze33ff) (+) (B) | SAFI3 B  (standard) | [*Acinetobacter nosocomialis*](https://docs.google.com/document/d/13sR9l8TvqBdnMS5XbT1vdYjt-tTESQPp/edit#heading=h.rl5p95jezrkg) | [1.99](https://docs.google.com/document/d/13sR9l8TvqBdnMS5XbT1vdYjt-tTESQPp/edit#heading=h.fx984lxbj9qx) | [*Acinetobacter nosocomialis*](https://docs.google.com/document/d/13sR9l8TvqBdnMS5XbT1vdYjt-tTESQPp/edit#heading=h.4znm6cahu39u) | [1.84](https://docs.google.com/document/d/13sR9l8TvqBdnMS5XbT1vdYjt-tTESQPp/edit#heading=h.fx984lxbj9qx) |
| [D9](https://docs.google.com/document/d/13sR9l8TvqBdnMS5XbT1vdYjt-tTESQPp/edit#heading=h.m6i3cr9opqgr) (+) (B) | TFG A  (standard) | [*Enterobacter kobei*](https://docs.google.com/document/d/13sR9l8TvqBdnMS5XbT1vdYjt-tTESQPp/edit#heading=h.uqvuv1c7v39l) | [1.94](https://docs.google.com/document/d/13sR9l8TvqBdnMS5XbT1vdYjt-tTESQPp/edit#heading=h.fx984lxbj9qx) | [*Enterobacter asburiae*](https://docs.google.com/document/d/13sR9l8TvqBdnMS5XbT1vdYjt-tTESQPp/edit#heading=h.mkqiybcq5rnn) | [1.75](https://docs.google.com/document/d/13sR9l8TvqBdnMS5XbT1vdYjt-tTESQPp/edit#heading=h.fx984lxbj9qx) |
| [D10](https://docs.google.com/document/d/13sR9l8TvqBdnMS5XbT1vdYjt-tTESQPp/edit#heading=h.aafb94tf3vj0) (+) (B) | TFG B  (standard) | [*Escherichia coli*](https://docs.google.com/document/d/13sR9l8TvqBdnMS5XbT1vdYjt-tTESQPp/edit#heading=h.k30nnnax91uw) | [1.75](https://docs.google.com/document/d/13sR9l8TvqBdnMS5XbT1vdYjt-tTESQPp/edit#heading=h.fx984lxbj9qx) | No Organism Identification Possible | [1.60](https://docs.google.com/document/d/13sR9l8TvqBdnMS5XbT1vdYjt-tTESQPp/edit#heading=h.fx984lxbj9qx) |
| [D11](https://docs.google.com/document/d/13sR9l8TvqBdnMS5XbT1vdYjt-tTESQPp/edit#heading=h.9fem06im3w6) (+) (B) | TFG C  (standard) | [*Klebsiella pneumoniae*](https://docs.google.com/document/d/13sR9l8TvqBdnMS5XbT1vdYjt-tTESQPp/edit#heading=h.snknyjj68yuc) | [1.95](https://docs.google.com/document/d/13sR9l8TvqBdnMS5XbT1vdYjt-tTESQPp/edit#heading=h.fx984lxbj9qx) | [*Klebsiella variicola*](https://docs.google.com/document/d/13sR9l8TvqBdnMS5XbT1vdYjt-tTESQPp/edit#heading=h.xac4jnesf49c) | [1.93](https://docs.google.com/document/d/13sR9l8TvqBdnMS5XbT1vdYjt-tTESQPp/edit#heading=h.fx984lxbj9qx) |
| [D12](https://docs.google.com/document/d/13sR9l8TvqBdnMS5XbT1vdYjt-tTESQPp/edit#heading=h.vtbxflim06eg) (+) (B) | 2CI  (standard) | *Escherichia coli* | [1.](https://docs.google.com/document/d/13sR9l8TvqBdnMS5XbT1vdYjt-tTESQPp/edit#heading=h.fx984lxbj9qx)97 | *Escherichia coli* | [1.](https://docs.google.com/document/d/13sR9l8TvqBdnMS5XbT1vdYjt-tTESQPp/edit#heading=h.fx984lxbj9qx)93 |
| [E1](https://docs.google.com/document/d/13sR9l8TvqBdnMS5XbT1vdYjt-tTESQPp/edit#heading=h.u2vfjn273am1) (+++) (A) | SAFG B  (standard) | [*Klebsiella pneumoniae*](https://docs.google.com/document/d/13sR9l8TvqBdnMS5XbT1vdYjt-tTESQPp/edit#heading=h.7f69eol1axqu) | [2.46](https://docs.google.com/document/d/13sR9l8TvqBdnMS5XbT1vdYjt-tTESQPp/edit#heading=h.fx984lxbj9qx) | [*Klebsiella pneumoniae*](https://docs.google.com/document/d/13sR9l8TvqBdnMS5XbT1vdYjt-tTESQPp/edit#heading=h.snknyjj68yuc) | [2.40](https://docs.google.com/document/d/13sR9l8TvqBdnMS5XbT1vdYjt-tTESQPp/edit#heading=h.fx984lxbj9qx) |
| [E2](https://docs.google.com/document/d/13sR9l8TvqBdnMS5XbT1vdYjt-tTESQPp/edit#heading=h.i7h7nqh48bs6) (+++) (A) | SAFG C  (standard) | [*Klebsiella pneumoniae*](https://docs.google.com/document/d/13sR9l8TvqBdnMS5XbT1vdYjt-tTESQPp/edit#heading=h.snknyjj68yuc) | [2.43](https://docs.google.com/document/d/13sR9l8TvqBdnMS5XbT1vdYjt-tTESQPp/edit#heading=h.fx984lxbj9qx) | [*Klebsiella pneumoniae*](https://docs.google.com/document/d/13sR9l8TvqBdnMS5XbT1vdYjt-tTESQPp/edit#heading=h.qc77odrrq8h3) | [2.43](https://docs.google.com/document/d/13sR9l8TvqBdnMS5XbT1vdYjt-tTESQPp/edit#heading=h.fx984lxbj9qx) |
| [E3](https://docs.google.com/document/d/13sR9l8TvqBdnMS5XbT1vdYjt-tTESQPp/edit#heading=h.bazsi9ipjf9r) (+++) (A) | TFG B  (standard) | [*Acinetobacter soli*](https://docs.google.com/document/d/13sR9l8TvqBdnMS5XbT1vdYjt-tTESQPp/edit#heading=h.khi7bzefr7rf) | [2.30](https://docs.google.com/document/d/13sR9l8TvqBdnMS5XbT1vdYjt-tTESQPp/edit#heading=h.fx984lxbj9qx) | [*Acinetobacter soli*](https://docs.google.com/document/d/13sR9l8TvqBdnMS5XbT1vdYjt-tTESQPp/edit#heading=h.9lg2f2oem36) | [2.28](https://docs.google.com/document/d/13sR9l8TvqBdnMS5XbT1vdYjt-tTESQPp/edit#heading=h.fx984lxbj9qx) |
| [E4](https://docs.google.com/document/d/13sR9l8TvqBdnMS5XbT1vdYjt-tTESQPp/edit#heading=h.v9vw2mww4s)  (-) (C) | DRFO A  (standard) | No Organism Identification Possible | [1.53](https://docs.google.com/document/d/13sR9l8TvqBdnMS5XbT1vdYjt-tTESQPp/edit#heading=h.fx984lxbj9qx) | No Organism Identification Possible | [1.51](https://docs.google.com/document/d/13sR9l8TvqBdnMS5XbT1vdYjt-tTESQPp/edit#heading=h.fx984lxbj9qx) |
| [E5](https://docs.google.com/document/d/13sR9l8TvqBdnMS5XbT1vdYjt-tTESQPp/edit#heading=h.lv2y2m4tg1e1) (+) (B) | DRFO B  (standard) | [*Klebsiella pneumoniae*](https://docs.google.com/document/d/13sR9l8TvqBdnMS5XbT1vdYjt-tTESQPp/edit#heading=h.snknyjj68yuc) | [1.71](https://docs.google.com/document/d/13sR9l8TvqBdnMS5XbT1vdYjt-tTESQPp/edit#heading=h.fx984lxbj9qx) | No Organism Identification Possible | [1.62](https://docs.google.com/document/d/13sR9l8TvqBdnMS5XbT1vdYjt-tTESQPp/edit#heading=h.fx984lxbj9qx) |
| [E6](https://docs.google.com/document/d/13sR9l8TvqBdnMS5XbT1vdYjt-tTESQPp/edit#heading=h.83pg46lyjshs) (+++) (A) | DRFO C  (standard) | [*Klebsiella pneumoniae*](https://docs.google.com/document/d/13sR9l8TvqBdnMS5XbT1vdYjt-tTESQPp/edit#heading=h.snknyjj68yuc) | [2.18](https://docs.google.com/document/d/13sR9l8TvqBdnMS5XbT1vdYjt-tTESQPp/edit#heading=h.fx984lxbj9qx) | [*Klebsiella pneumoniae*](https://docs.google.com/document/d/13sR9l8TvqBdnMS5XbT1vdYjt-tTESQPp/edit#heading=h.7f69eol1axqu) | [2.13](https://docs.google.com/document/d/13sR9l8TvqBdnMS5XbT1vdYjt-tTESQPp/edit#heading=h.fx984lxbj9qx) |
| [E7](https://docs.google.com/document/d/13sR9l8TvqBdnMS5XbT1vdYjt-tTESQPp/edit#heading=h.95tc223gdb21)  (-) (C) | MCI A  (standard) | No Organism Identification Possible | [1.53](https://docs.google.com/document/d/13sR9l8TvqBdnMS5XbT1vdYjt-tTESQPp/edit#heading=h.fx984lxbj9qx) | No Organism Identification Possible | [1.45](https://docs.google.com/document/d/13sR9l8TvqBdnMS5XbT1vdYjt-tTESQPp/edit#heading=h.fx984lxbj9qx) |
| [E8](https://docs.google.com/document/d/13sR9l8TvqBdnMS5XbT1vdYjt-tTESQPp/edit#heading=h.fa7oc62zs0eq) (+) (B) | MCI B  (standard) | [*Klebsiella variicola*](https://docs.google.com/document/d/13sR9l8TvqBdnMS5XbT1vdYjt-tTESQPp/edit#heading=h.rbu64r2ipdl6) | [1.73](https://docs.google.com/document/d/13sR9l8TvqBdnMS5XbT1vdYjt-tTESQPp/edit#heading=h.fx984lxbj9qx) | No Organism Identification Possible | [1.67](https://docs.google.com/document/d/13sR9l8TvqBdnMS5XbT1vdYjt-tTESQPp/edit#heading=h.fx984lxbj9qx) |
| [E9](https://docs.google.com/document/d/13sR9l8TvqBdnMS5XbT1vdYjt-tTESQPp/edit#heading=h.ated9f67pgs2) (+) (B) | MCI C  (standard) | [*Klebsiella variicola*](https://docs.google.com/document/d/13sR9l8TvqBdnMS5XbT1vdYjt-tTESQPp/edit#heading=h.7xzgpnbtsfgm) | [1.77](https://docs.google.com/document/d/13sR9l8TvqBdnMS5XbT1vdYjt-tTESQPp/edit#heading=h.fx984lxbj9qx) | [*Klebsiella pneumoniae*](https://docs.google.com/document/d/13sR9l8TvqBdnMS5XbT1vdYjt-tTESQPp/edit#heading=h.snknyjj68yuc) | [1.75](https://docs.google.com/document/d/13sR9l8TvqBdnMS5XbT1vdYjt-tTESQPp/edit#heading=h.fx984lxbj9qx) |
| [E10](https://docs.google.com/document/d/13sR9l8TvqBdnMS5XbT1vdYjt-tTESQPp/edit#heading=h.pddq6vniojb9) (+++) (A) | SAFG A  (standard) | [*Klebsiella pneumoniae*](https://docs.google.com/document/d/13sR9l8TvqBdnMS5XbT1vdYjt-tTESQPp/edit#heading=h.7f69eol1axqu) | [2.00](https://docs.google.com/document/d/13sR9l8TvqBdnMS5XbT1vdYjt-tTESQPp/edit#heading=h.fx984lxbj9qx) | [*Klebsiella pneumoniae*](https://docs.google.com/document/d/13sR9l8TvqBdnMS5XbT1vdYjt-tTESQPp/edit#heading=h.snknyjj68yuc) | [1.97](https://docs.google.com/document/d/13sR9l8TvqBdnMS5XbT1vdYjt-tTESQPp/edit#heading=h.fx984lxbj9qx) |
| [E11](https://docs.google.com/document/d/13sR9l8TvqBdnMS5XbT1vdYjt-tTESQPp/edit#heading=h.r4ifh3cqu0nn) (+) (B) | SACI  (standard) | *Citrobacter braaki* | [1.](https://docs.google.com/document/d/13sR9l8TvqBdnMS5XbT1vdYjt-tTESQPp/edit#heading=h.fx984lxbj9qx)86 | *Citrobacter braaki* | [1.](https://docs.google.com/document/d/13sR9l8TvqBdnMS5XbT1vdYjt-tTESQPp/edit#heading=h.fx984lxbj9qx)72 |
| [E12](https://docs.google.com/document/d/13sR9l8TvqBdnMS5XbT1vdYjt-tTESQPp/edit#heading=h.n5pxluhneq38) (-) (C) | SCGC B  (standard) | no peaks found | [0.00](https://docs.google.com/document/d/13sR9l8TvqBdnMS5XbT1vdYjt-tTESQPp/edit#heading=h.fx984lxbj9qx) | no peaks found | [0.00](https://docs.google.com/document/d/13sR9l8TvqBdnMS5XbT1vdYjt-tTESQPp/edit#heading=h.fx984lxbj9qx) |
| [F1](https://docs.google.com/document/d/13sR9l8TvqBdnMS5XbT1vdYjt-tTESQPp/edit#heading=h.73kq9nlr358z) (+) (B) | SCGC C  (standard) | [*Enterobacter kobei*](https://docs.google.com/document/d/13sR9l8TvqBdnMS5XbT1vdYjt-tTESQPp/edit#heading=h.y28a4iafwixn) | [1.79](https://docs.google.com/document/d/13sR9l8TvqBdnMS5XbT1vdYjt-tTESQPp/edit#heading=h.fx984lxbj9qx) | [*Enterobacter roggenkampii*](https://docs.google.com/document/d/13sR9l8TvqBdnMS5XbT1vdYjt-tTESQPp/edit#heading=h.47uyf78r30y5) | [1.72](https://docs.google.com/document/d/13sR9l8TvqBdnMS5XbT1vdYjt-tTESQPp/edit#heading=h.fx984lxbj9qx) |
| [F2](https://docs.google.com/document/d/13sR9l8TvqBdnMS5XbT1vdYjt-tTESQPp/edit#heading=h.37g6fnobrbyt)  (+++) (A) | SACI A  (standard) | *Escherichia coli* | 2.56 | *Escherichia coli* | 2.22 |
| [F3](https://docs.google.com/document/d/13sR9l8TvqBdnMS5XbT1vdYjt-tTESQPp/edit#heading=h.l1l90gy1pvck) (+++) (A) | MCOA A  (standard) | [*Klebsiella pneumoniae*](https://docs.google.com/document/d/13sR9l8TvqBdnMS5XbT1vdYjt-tTESQPp/edit#heading=h.snknyjj68yuc) | [2.05](https://docs.google.com/document/d/13sR9l8TvqBdnMS5XbT1vdYjt-tTESQPp/edit#heading=h.fx984lxbj9qx) | [*Klebsiella pneumoniae*](https://docs.google.com/document/d/13sR9l8TvqBdnMS5XbT1vdYjt-tTESQPp/edit#heading=h.qc77odrrq8h3) | [1.94](https://docs.google.com/document/d/13sR9l8TvqBdnMS5XbT1vdYjt-tTESQPp/edit#heading=h.fx984lxbj9qx) |
| [F4](https://docs.google.com/document/d/13sR9l8TvqBdnMS5XbT1vdYjt-tTESQPp/edit#heading=h.nakwwthoaj01) (+) (B) | MCOA B  (standard) | [*Klebsiella pneumoniae*](https://docs.google.com/document/d/13sR9l8TvqBdnMS5XbT1vdYjt-tTESQPp/edit#heading=h.snknyjj68yuc) | [1.77](https://docs.google.com/document/d/13sR9l8TvqBdnMS5XbT1vdYjt-tTESQPp/edit#heading=h.fx984lxbj9qx) | No Organism Identification Possible | [1.57](https://docs.google.com/document/d/13sR9l8TvqBdnMS5XbT1vdYjt-tTESQPp/edit#heading=h.fx984lxbj9qx) |
| [F5](https://docs.google.com/document/d/13sR9l8TvqBdnMS5XbT1vdYjt-tTESQPp/edit#heading=h.gnh9r58oprwv) (+) (B) | SCI A  (standard) | [*Klebsiella pneumoniae*](https://docs.google.com/document/d/13sR9l8TvqBdnMS5XbT1vdYjt-tTESQPp/edit#heading=h.snknyjj68yuc) | [1.78](https://docs.google.com/document/d/13sR9l8TvqBdnMS5XbT1vdYjt-tTESQPp/edit#heading=h.fx984lxbj9qx) | No Organism Identification Possible | [1.51](https://docs.google.com/document/d/13sR9l8TvqBdnMS5XbT1vdYjt-tTESQPp/edit#heading=h.fx984lxbj9qx) |
| F6 (+++)(A) | SFO 10-1  A | *Salmonella* spp*.* | 2.38 | *Salmonella* spp. | 2.02 |
